# Following the Mixtures of Organic Micropollutants with *In Vitro* Bioassays in a Large Lowland River from Source to Sea

**DOI:** 10.1021/acsenvironau.4c00059

**Published:** 2025-01-19

**Authors:** Elena Hommel, Maria König, Georg Braun, Martin Krauss, Norbert Kamjunke, Werner Brack, Anna Matousu, Tina Sanders, Ingeborg Bussmann, Eric P. Achterberg, Björn Raupers, Beate I. Escher

**Affiliations:** † Department of Cell Toxicology, Helmholtz Centre for Environmental Research−UFZ, Permoserstr. 15, Leipzig 04318, Germany; ‡ Department Exposure Science, Helmholtz Centre for Environmental Research − UFZ, Permoserstr. 15, Leipzig 04318, Germany; § Department of River Ecology, Helmholtz Centre for Environmental Research − UFZ, Brückstr. 3a, Magdeburg 39114, Germany; ∥ Institute of Ecology, Evolution and Diversity - Goethe University, Max-von-Laue-Str. 13, Frankfurt am Main 60438, Germany; ⊥ Biology Centre, Czech Academy of Sciences, Institute of Hydrobiology, Ceské Budejovice 370 05, Czech Republic; # Institute of Carbon Cycles, Helmholtz Centre Hereon, Max-Planck-Straße 1, Geesthacht 21502, Germany; ∇ Department of Shelf Sea System Ecology, Alfred-Wegener-Institut, Helmholtz Zentrum für Polar- und Meeresforschung, Kurpromenade 201, Helgoland 27498, Germany; ○ GEOMAR, Helmholtz Centre for Ocean Research Kiel, Wischhofstraße 1-3, Kiel 24148, Germany; ◆ Environmental Toxicology, Department of Geosciences, Eberhard Karls University Tübingen, Schnarrenbergerstr. 94-96, Tübingen 72076, Germany

**Keywords:** bioassay, mixture toxicity, water quality monitoring, estrogenicity, neurotoxicity, oxidative stress, xenobiotic metabolism

## Abstract

Human-impacted rivers often contain a complex mixture of organic micropollutants, including pesticides, pharmaceuticals and industrial compounds, along with their transformation products. Combining chemical target analysis for exposure with *in vitro* bioassays for effect assessment offers a holistic view of water quality. This study targeted the River Elbe in Central Europe, known for its anthropogenic pollution exposure, to obtain an inventory of micropollutant contamination during base flow and to identify hotspots of contamination. We identified tributaries as sources of chemicals activating the aryl hydrocarbon receptor quantified with the AhR-CALUX assay, including historically contaminated tributaries and a newly identified Czech tributary. Increased neurotoxicity, detected by differentiated SH-SY5Y neurons’ cytotoxicity and shortened neurite length, was noted in some Czech tributaries. A hotspot for chemicals activating the oxidative stress response in the AREc32 assay was found in the middle Elbe in Germany. An increase in oxidative stress inducing chemicals was observed in the lower Elbe. While effect-based trigger values (EBT) for oxidative stress response, xenobiotic metabolism and neurotoxicity were not exceeded, estrogenicity levels surpassed the EBT in 14% of surface water samples, posing a potential threat to fish reproduction. Target analysis of 713 chemicals resulted in the quantification of 487 micropollutants, of which 133 were active in at least one bioassay. Despite this large number of bioactive quantified chemicals, the mixture effects predicted by the concentrations of the quantified bioactive chemicals and their relative effect potency explained only 0.002–1.2% of the effects observed in the surface water extracts, highlighting a significant unknown fraction in the chemical mixtures. This case study established a baseline for understanding pollution dynamics and spatial variations in the Elbe River, offering a comprehensive view of potential chemical effects in the water and guiding further water quality monitoring in European rivers.

## Graphical abstract

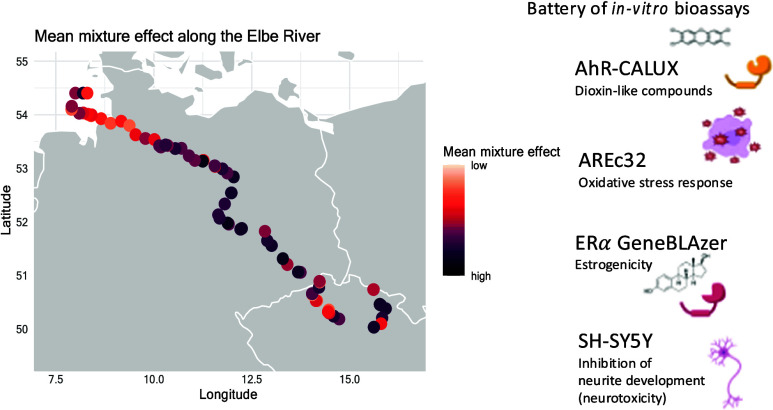

## Introduction

The Elbe River is a critical water resource in Europe, serving multiple functions such as provision of drinking and irrigation water, and sustaining diverse ecosystems. However, human influences, including shipping, discharges from wastewater treatment plants (WWTP), historical pollution and runoff from urban and agricultural areas, have led to chemical pollution in the Elbe. This pollution poses significant risks, including biodiversity loss,^1^ degradation of drinking water quality and impairment of ecosystem services.^2^ A particular concern is the presence of organic micropollutants such as pharmaceuticals, pesticides and industrial compounds at low concentrations in the nano- to microgram per liter range. These micropollutants, which are present in complex chemical mixtures, show a diverse composition, varying in persistence and toxicological effects. Their number will further increase when they degrade or transform into various byproducts.^3^

Traditional *in vivo* toxicity testing methods used in risk assessments raise ethical concerns and face practical and scalability issues. Consequently, new approach methodologies are under investigation for assessing the toxicity and risk of chemicals. Cell-based *in vitro* bioassays offer a promising, cost-effective alternative for evaluating the effects of complex environmental mixtures in a high-throughput approach. These bioanalytical tools complement chemical analyses by providing insights into environmentally relevant toxicity end points and can account for the mixture effects of known and unknown compounds.^3^ This approach can expedite risk assessment processes and reduce the reliance on animal testing.^3^ Typically, it is not sufficient to use a single *in vitro* bioassay but it is recommended to apply a battery of bioassays that cover different groups of environmentally relevant modes of action (MoA), among them endocrine effects, interferences with metabolism, reactive toxicity and adaptive stress responses.^4^ Bioassay test batteries are commonly applied to assess wastewater treatment efficacy,^5^ drinking water treatment efficacy,^6^ and changes in water quality during storm events^7^ or harmful algae blooms.^8^

In this study, we employed a battery of reporter gene assays that has been demonstrated to be suitable for assessment of wastewater and surface water quality to investigate the presence of organic pollutants in the River Elbe and assess their baseline toxicological response. The test battery included the AhR-CALUX assay for aryl hydrocarbon receptor (AhR) activation and xenobiotic metabolism,^9^ AREc32 for oxidative stress response,^10^ and ERα-GeneBLAzer for estrogen receptor (ER) activation.^11^ In addition, a neurotoxicity assay based on neurite outgrowth inhibition in differentiated SH-SY5Y cells was applied.^12^ With this bioanalytical test battery, we aimed at conducting a comprehensive toxicological assessment of the entire Elbe River, from its source to its estuary. For the section in Germany from the Czech border to the beginning of the estuary a Lagrangian sampling approach was employed. This approach, which involves tracking a water parcel, allows us to observe how the presence and effects of contaminants vary along the river without the impact of temporal variation, particularly with inputs from WWTPs and tributaries.

## Materials and Methods

### Study Area and Sampling

The Elbe River flows through Central Europe and drains a catchment of 148,268 km^2^ with 25 million inhabitants. The sampling encompassed a comprehensive collection of samples along the entire river starting from its origin in the Czech Republic to the German Bight, with an overview of all sampling sites shown Table S1 and Figure S1. In the Czech part a Lagrangian sampling approach was not possible as approximately 200 km of the river is intersected by 25 weirs with locks, which impedes a reasonable estimation of flow velocities. Therefore, grab samples were taken along the river and its tributaries. Starting from the Czech/German border near Schmilka down to Geesthacht, a Lagrangian sampling approach was applied using the research vessel Albis, which sampled nearly the same water package along the river downstream according to its travel time. At each site, lateral samples were taken in the middle and either side of the river. At WWTPs, 24-h composite samples were taken using automated samplers; the effluent sample periods were chosen to span around the time point the corresponding river water samples were taken, the influent samples were taken earlier to account approximately for the hydraulic retention time in the WWTP. Samples were stored at 4 °C after they were taken and frozen at −20 °C in the evening until further processing.

The sampling period was from the 27 June to 14 September 2023. In the tidal Elbe, the sampling strategy shifted to a steady-state approach, conducting sampling over 2 days against the water flow at ebb tide using the research vessel L. Prandtl.^13^ This was done against the draining flood from the island Scharhörn, 20 km offshore of Cuxhaven, toward the weir Geesthacht between August 23–25, 2023. Sampling in the German Bight was performed with the research vessel RV Littorina (Stern cruise number 10–2, September 2–9, 2023) and Mya II (Stern cruise number 10–3, September 11–14, 2023).^14^ Surface water was sampled using a CTD rosette, filled into the prepared polypropylene bottles and stored at 4 ± 1 °C.

Further details of the sampling campaign can be found in the expedition report.^14^ In total 133 water samples were collected (in the excel file Table SE1), including 16 tributaries contributing at least 1% of the discharge to the Elbe at dry weather conditions and 10 WWTP influents and effluents, the latter contributing at least 0.1% to the discharge. River water sampling sites were located at least 4 km downstream of a WWTP inflow into the Elbe except for the Meissen WWTP, where the Zehren site is approximately 1.5 km downstream. Field blank samples were prepared by adding 150 mL of LC-MS grade water into polypropylene bottles, which were traveling along the sample bottles for the whole sampling campaign.

### Sample Extraction

Solid phase extraction (SPE) using an automated device (Promochrom SPE-03) and Chromabond HR-X cartridges (6 mL, 200 mg sorbent, from Macherey-Nagel, Düren, Germany) based on the method described in Maurer et al.^15^ was used to extract and enrich micropollutants from 650 mL of Elbe river water, tidal and North Sea water, wastewater treatment plant influent or effluent and field blanks (Table S2). In parallel, processing blanks using 150 mL of LC-grade water were also filtered. The SPE extracts were blown down in a nitrogen stream and redissolved in methanol to an extraction factor (EF; L_water_/L_extract_) of 1000 (see Supporting Information, section S2). Blank samples were processed the same way.

### Chemical Analysis and Compound Quantification

The SPE extract of surface water and WWTP samples were analyzed by a target screening method using liquid chromatography high-resolution mass spectrometry (LC–HRMS) and a quadrupole-Orbitrap MS (Exploris 480, Thermo Scientific). The analyte list included 713 chemicals that are recognized or suspected pollutants in surface waters.^16^ Quantification was done using method-matched internal standard calibration employing 40 isotope-labeled internal standards. For quantification, we used a workflow combining peak detection in MZmine 2,^17^ and the R package MZquant (https://git.ufz.de/wana_public/mzquant^18^) as well as the Tracefinder 5.1 software from Thermo Fisher Scientific. For details, see Supporting Information, section S3. As has been previously shown,^19^ SPE provides a suitable sample preparation approach for chemical target screening, and can be effectively applied for sample preparation for effect analysis.

### Bioanalysis

The CellSensor ERα-GeneBLAzer cells were obtained from Thermo Fisher Scientific,^20^ SH-SY5Y from Sigma (94030304), AREc32 cells by courtesy of C. Roland Wolf, Cancer Research U.K.,^21^ and AhR-CALUX cells by courtesy of Michael Denison, UC Davis.^9^ Protocols for cell cultivation, differentiation and quality assurance of the selected bioassays (AhR-CALUX, AREc32, ERα GeneBLAzer and SH-SY5Y) are detailed elsewhere (summary in Supporting Information Text S4, and Tables S3–S5).^22,23^ Cell viability was assessed in parallel to effect measurement based on cell confluency using an IncuCyte S3 live cell imaging system (Essen BioScience, Ann Arbor, Michigan) or live/dead cell staining for SH-SY5Y cells. Concentrations reducing cell viability by 10% or more were excluded from further data evaluation to avoid artifacts due to the cytotoxic burst effect.^24^

Cells were exposed to 11 different concentrations in serial dilution, with the highest relative extraction factor (REF, L_water_/L_bioassay_) of 100 for surface water, 50 for WWTP effluents and 25 for WWTP influents. The REF takes the enrichment during the extraction procedure (the extraction factor EF) and the dilution in the assay (dilution factor DF) into consideration (REF = EF × DF). The experimental workflow spanned 3 days: cell seeding (day 1), sample dosing (day 2), and cytotoxicity and effect detection (day 3). Thirty μL of cell suspension were seeded in a 384 well plate using a MultiFlow dispenser from Biotek (5000 cells/well for ERα GeneBLAzer in a black PDL coated plate, 3100 cells/well for SH-SY5Y in a black Col-I coated plate, 3250 cells/well for AhR-CALUX in a white PDL coated plate and 2650 cells/well for AREc32 in a white TC treated plate). Plates were incubated for 24 h at 37 °C, 5% CO_2_ and 100% humidity to allow cell attachment. Before dosing the chemicals to the cells, the confluency of the cells was measured again using the IncuCyte S3 described above.

Stock solutions of reference chemicals were prepared in methanol (purity ≥99%, LC-MS grade) at low and high concentration ranges, overlapping at 50% effect in the dose–response curve for each plate measured as the positive control. The chemicals used as reference compounds and their concentration range in the *in vitro* assays are listed in Table S6. The last two rows of the 384 well plate remained without sample application and were used as the negative control. To eliminate solvent effects, appropriate aliquots of methanolic extracts were evaporated to dryness and reconstituted in 120 μL assay medium before bioanalysis. Sample application and subsequent serial dilution were performed using an automated liquid handling robot (Hamilton MICROLAB Star). After 24 h incubation at 37 °C, 5% CO_2_ and 100% relative humidity, cell confluence was measured again using the Incucyte S3. For the SH-SY5Y assay, viability was measured by staining with nuclear green and propidium iodide as described in Lee et al.^12^ Reporter gene activation or morphological changes of the cells were measured according to established protocols (see Supporting Information Text S4).

### Data Evaluation

Nonlinear regression was utilized to generate a concentration–response curve (CRC) that fits the reference compound data, setting the maximum response of the reference compound to 100% effect and adjusting sample values accordingly. The effect concentration (EC) indicates the concentration of a chemical that triggers a defined response, such as reaching 10% of the maximum effect (EC_10_). All ECs and cytotoxic inhibition concentrations (ICs) were expressed in units of REF.

Typically, CRCs exhibit a linear trend up to 30% absolute effect, which allows for simplified linear regression.^25^ A cutoff of 30% was applied for all assays to focus on the lower portion of the CRC. For the AhR, AREc32 and ERα the linear model was used to calculate the EC_10_ or EC_IR1.5_. Notably, the log–logistic evaluation was deemed more accurate for the SH-SY5Y assay, as well as for determining the IC_10_ in the AREc32 assay.

At elevated concentrations, significant effects such as cytotoxicity may arise, particularly in complex environmental mixtures containing numerous weakly acting chemicals. In such scenarios, cytotoxicity can mask specific effects, making them difficult to discern. The concentration at which cell viability decreases by 10% is termed the inhibition concentration of 10% (IC_10_), calculated using a formula derived from the slope of the linear range of the CRC (eq 1). The standard error (SE) of the IC_10_ was determined via error propagation using eq 2. The EC values and their SE can also be calculated using eqs 1 and 2, respectively.

$${\mathrm{IC}}_{10}=\frac{0.1}{\mathrm{slope}}\;\mathrm{and}\;{\mathrm{EC}}_{10}=\frac{0.1}{\mathrm{slope}}\;\mathrm{or}\;{\mathrm{EC}}_{\mathrm{IR}1.5}=\frac{0.5}{\mathrm{slope}}$$ 1

$$\mathrm{SE}\left({\mathrm{IC}}_{10}\right)=\frac{0.1}{\mathrm{slop}e^{2}}\cdot \mathrm{SE}\left(\mathrm{slope}\right)\;\mathrm{and}\;\mathrm{SE}\left({\mathrm{EC}}_{10}\right)=\frac{0.1}{\mathrm{slop}e^{2}}\cdot \mathrm{SE}\left(\mathrm{slope}\right)\;\mathrm{or}\;\mathrm{SE}\left({\mathrm{EC}}_{\mathrm{IR}1.5}\right)=\frac{0.5}{\mathrm{slop}e^{2}}\cdot \mathrm{SE}\left(\mathrm{slope}\right)$$ 2

Since the oxidative stress response is not a receptor-mediated effect, no maximum effect could be determined for the reference compound. Instead, the induction ratio (IR) was calculated for each sample which is defined as the ratio of the signal from the sample in relative light units (RLU) to the signal from the unexposed cells (eq 3).

3 $$i\mathrm{nduction}\;r\mathrm{atio}\;\left(\mathrm{IR}\right)=\frac{\mathrm{RL}U_{\mathrm{sample}}}{\frac{\sum_{i=1}^{n}\mathrm{RL}U_{\mathrm{unexposed cells}}}{n}}$$

The concentration which leads to an IR of 1.5 is called the EC_IR1.5_ and is the activity benchmark in the AREc32 assay. The EC_IR1.5_ is calculated from the linear model of IR versus concentration, utilizing concentrations below cytotoxic levels (eq 1). The standard error of the EC_IR1.5_ was determined using eq 2.

The data evaluation process was automated using R software (version 4.3.1). Linear regression and log–logistic models were employed for each sample and reference compound. The R scripts used for this analysis, along with detailed explanations of the data processing steps, can be found on GitLab: https://git.ufz.de/braung/automatedbioassayscreening (version from 01.04.2024). A four parameter log–logistic concentration response model was used for the calculation of IC_10_ and EC_10_ corresponding to the absolute 10% effect using the tcpl R package.^26^ Concentrations above IC_10_ of cytotoxicity were excluded from the linear CRC of the reporter gene activation for the derivation of EC_10_ and EC_IR1.5_.^25^ Additionally, a variable to characterize the goodness-of-fit of the CRC was determined. This confidence variable was calculated using the standard error of the EC_10_ or IC_10_ and the R-squared value of the linear regression, with a threshold of 0.7 (eq 4). If the confidence variable fell below the threshold, manual evaluation of the curve and fitting was performed, and the data were included if deemed appropriate after expert assessment.

4 $$c\mathrm{onfidence}=\left(\frac{1-\mathrm{SE}\left({\mathrm{EC}}_{10}/{\mathrm{IC}}_{10}\right)}{{\mathrm{EC}}_{10}/{\mathrm{IC}}_{10}}\right)\cdot R^{2}$$

### Water Quality Assessment and Specificity Analysis

Concentration addition is a common model for predicting the effects of complex chemical mixtures.^7^ Assuming concentration addition, the mixture components contribute additively to the overall effect by acting through the same MoA or biological pathway.^3^ For better comparison between assays and samples, bioanalytical equivalent concentrations (BEQ_bio_) were used to express the potency of a single chemical or complex mixtures in terms of an equivalent concentration of the reference compound that produces the same biological response. This additive mixture model is valid for many *in vitro* and *in vivo* assays.^7,12,27–31^ The BEQ_bio_ (in units of ng of reference compound per liter of sampled water) can be calculated by dividing the EC_10_ of the reference compound (see Table SE2) by the EC_10_ of the water sample (Table SE3, see eq 5, with corresponding standard error calculation using error propagation eq 6 ^3^).

5 $${\mathrm{BEQ}}_{\mathrm{bio}}=\frac{{\mathrm{EC}}_{10\;\mathrm{reference}}}{{\mathrm{EC}}_{10\;\mathrm{sample}}}$$

6 $$SE_{\mathrm{BEQ}}=\sqrt{\frac{1}{{\mathrm{EC}}_{10\;\mathrm{sample}}^{2}}\cdot SE^{2}\left({\mathrm{EC}}_{10\;\mathrm{reference}}\right))+\frac{{\mathrm{EC}}_{10\;\mathrm{reference}}^{2}}{{\mathrm{EC}}_{10\;\mathrm{sample}}^{4}}\cdot SE^{2}\left({\mathrm{EC}}_{10\;\mathrm{sample}}\right)}$$

In the AhR and AREc32 assays, B­[*a*]­P-EQ (benzo­[*a*]­pyrene) and Dichlorvos-EQ were used instead of 2,3,7,8-tetrachlorodibenzodioxin equivalent concentrations (TCDD-EQ) and *tert*-butylhydroquinone equivalent concentrations (tBHQ-EQ) because the quality control compounds would not occur in environmental water samples.

*In vitro* bioassays are highly sensitive and may detect a signal in “clean” waters, especially if they have been enriched. Hence, not every bioassay response implies that there will be an associated ecotoxicological risk. To evaluate the chemical water quality the calculated BEQ_bio_ values are compared with effect based-trigger values (EBT), which were derived by reading across from environmental quality standards of the European Union Water Framework Directive,^32^ and by considering mixture effects.^33^ The EBT is an assay-specific threshold that differentiates whether a mixture is likely to produce adverse effects and is used to protect the aquatic ecosystem health and exposed aquatic organisms.^3^

The specificity ratio (SR_cytotoxicity_) serves as a measure of a chemical’s selectivity in a given bioassay. It is calculated by comparing the experimental cytotoxicity (IC_10_) to the effect concentration (EC_10_ or EC_IR1.5_), as shown in eq 7.

7 $$SR_{\mathrm{cytotoxicity}}=\frac{{\mathrm{IC}}_{10}}{{\mathrm{EC}}_{10}}$$

The SR_cytotoxicity_ helps determine whether the observed effect is selective or if it is accompanied by cytotoxicity. A higher SR_cytotoxicity_ suggests that the chemical’s MoA is more specific to a particular end point rather than affecting overall cell viability. This approach has been applied in other studies investigating end points such as hormone receptor activation, oxidative stress response and neurite outgrowth inhibition.^23,31^

### Iceberg Modeling

Iceberg modeling links the measured effects in the bioassays with predicted effects based on quantified concentrations from the target analysis.^3^ The BEQ_bio_ from the bioassay measurements of the samples which capture the entire mixture effect was compared with the predicted effect (BEQ_chem_). The BEQ_chem_ was derived from the EC values of the detected chemicals based on chemical analysis and the application of mixture models. To calculate BEQ_chem_, first the EC ratio of the reference compound and chemical *i* was calculated to give the relative effect potency for each chemical *i* (REP_*i*_; eq 8). Second, the REP_*i*_ was multiplied by the detected concentration (*C*_*i*_) of chemical *i* to calculate BEQ_*i*_ for individual chemicals. Then, the BEQ_chem_ for the whole sample was calculated by summing up BEQ_*i*_ for all detected chemicals (eq 9).

8 $$\mathrm{RE}P_{i}=\frac{{\mathrm{EC}}_{10}\left(\mathrm{reference chemical}\right)}{EC_{10}\left(i\right)}$$

9 $$\mathrm{BE}Q_{\mathrm{chem}}=\sum_{i=1}^{n}\mathrm{BE}Q_{i}=\sum_{i=1}^{n}\mathrm{RE}P_{i}\cdot C_{i}$$

The iceberg modeling was performed for the bioassays SH-SY5Y, AREc32 and AhR-CALUX, using a workflow developed in R. By comparison of BEQ_chem_ with BEQ_bio_ for each bioassay, the contribution of the individual detected chemical *i* to the overall mixture effect can be quantified (eq 10). The contribution of individual detected chemical *i* to the BEQ_chem_ is defined by eq 11.

$$\%\;\mathrm{effect explained}=\frac{\mathrm{BE}Q_{\mathrm{chem}}}{\mathrm{BE}Q_{\mathrm{bio}}}\cdot 100\%$$ 10

$$\%\;\mathrm{contribution of chemical}\;i\;\mathrm{to BE}Q_{\mathrm{chem}}=\frac{\mathrm{BE}Q_{i}}{\mathrm{BE}Q_{\mathrm{chem}}}\cdot 100\%$$ 11

## Results and Discussion

### Bioanalysis

Reference compounds were run in all bioassay plates. The EC values of the reference compounds (Table SE2) passed the quality control criteria. A summary of the frequency of detected EC and IC_10_ of the water extracts for all bioassays is displayed in Table S7. For five samples in AREc32 EC_IR1.5_ values were higher than the IC_10_ values (Table S7) indicating that the effect is not independent but caused by cytotoxicity, rendering those EC_IR1.5_ values invalid. If close to cell death, numerous adaptive stress responses, among them the oxidative stress response, are triggered nonspecifically.^34,35^ Four samples in the neurotoxicity assay also showed EC_10_ > IC_10_. Evidently, if neurons are killed their neurites also disappear, so this is also not a specific effect. Estrogenic effects were masked by cytotoxicity for 40 of the 133 samples (Table S7). Additionally, while certain samples showed no observable effects within the tested concentration range (up to REF 100), inhibition of cell viability was observed in 101 (ERα) to 113 (AhR) samples (Table S7).

The 133 water samples exhibited a high diversity of effect patterns in the bioassays (Table SE3). Neurite outgrowth inhibition was often the most responsive end point (highest fraction of active samples 89%), followed by the responses of assays indicative of xenobiotic metabolism and activation of the AhR (76%). The oxidative stress response assay and activation of the ER showed the lowest fraction of active samples (both 66%). Comparing the different samples taken from the lateral sampling (left, middle and right side of the river) in the German part of the Elbe, some sampling points showed larger lateral variability than others (coefficient of variance (CV) range from 2.5 to 82.6). This is probably caused by incomplete mixing directly after point sources such as the tributaries. Nonetheless, the overall variation was statistically higher across the length of the river than across its width. The IC_10_ and EC values of the surface water samples ranged from REF 1 to 100 for 96% of the samples, which means that the water had to be enriched to cause 10% effect, an IR of 1.5 or 10% cytotoxicity. Only 26 out of 778 samples (3%) had IC_10_ or EC values between REF 0.1 to 1, which means that the sample had to be diluted to show 10% effect, an IR of 1.5 or 10% cytotoxicity. REF 100 was the highest tested concentration, any samples that did not cause any effects up to REF 100 were considered inactive. EC_10_ and IC_10_ were lowest (most toxic and potent) for WWTP influents, followed by WWTP effluents and then the surface waters. There were no clear trends between the Elbe main river and the tributaries. When the EC values were color-coded and plotted into the geospatial map of the Elbe (Figures S3, S5, S7, and S9), the effects tended to be more moderate close to the source and in the tidal Elbe and the German Bight.

The cytotoxicity data IC_10_ are plotted against the corresponding EC values in Figure 1. The diagonal lines in Figure 1 represent their SR_cytotoxicity_. SR_cytotoxicity_ < 1 would mean that the effect was masked by cytotoxicity and no EC values were reported in Table SE3 for AhR CALUX, AREc32 and ERα GeneBLAzer in such cases. Neurotoxicity is slightly different because cytotoxicity and neurite outgrowth inhibition appear at the same concentrations when effects are not specific but if neurite outgrowth inhibition occurs at lower concentrations, neurotoxicity is specific. High SR_cytotoxicity_ can have several causes, such as few highly specifically acting chemicals dominating the mixture effect or many chemicals with low SR_cytotoxicity_ acting together. SR_cytotoxicity_ close to 1 indicate that the effect is a secondary effect of the cytotoxicity and likely nonspecific.

**Figure 1:**
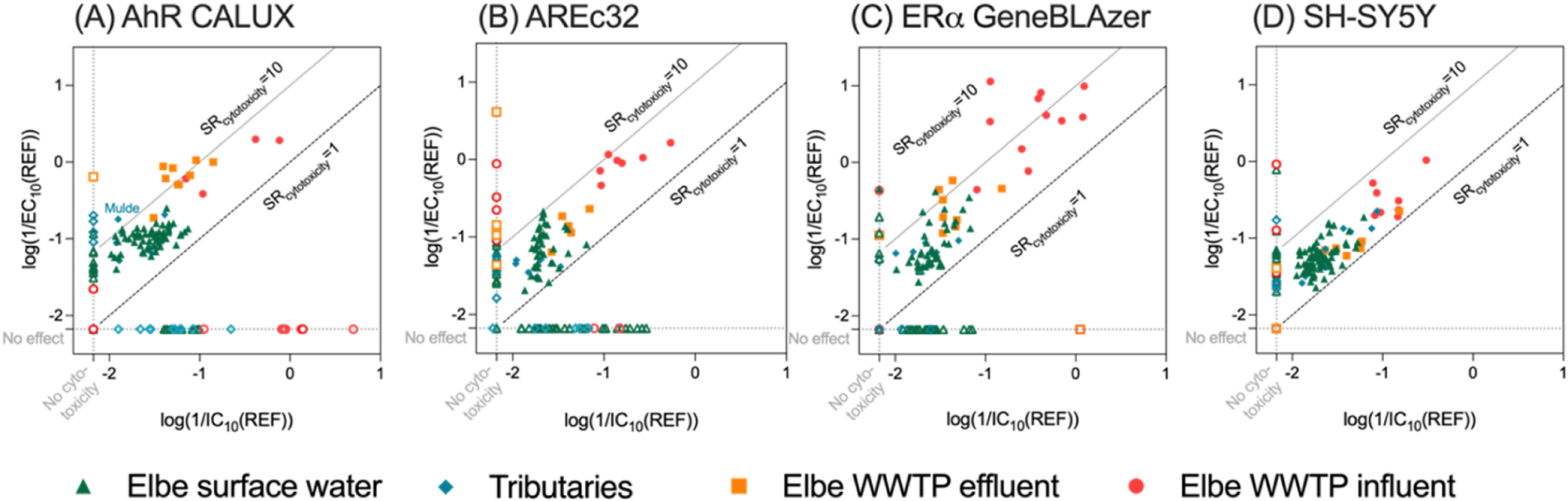
Inhibitory concentration (IC_10_) and effect concentration (EC_10_ or EC_IR1.5_) of (A) AhR CALUX; (B) AREC32, (C) ERα GeneBLAzer and (D) the neurotoxicity assays were plotted together on an inverted logarithmic scale to compare the degree of toxicity. Their ratio, that is the specificity ratio SR_cytotoxicity_, represents an indicator of the specificity of effects. Green triangles indicate surface water of the Elbe, blue diamonds the tributaries, orange squares the WWTP (wastewater treatment plant) effluents and red circles the WWTP influents. The SR_cytotoxicity_ of 1 and 10 are indicated by dotted lines.

WWTP effluent and influent extracts showed not only higher cytotoxicity and effects overall, but also more specific effects on AhR and ERα than surface water (SR_cytotoxicity_ > 10), whereas for AREc32 and SH-SY5Y WWTP and surface waters covered a lower range of SR_cytotoxicity_ (1 < SR_cytotoxicity_ < 10) despite the overall effects and cytotoxicity being substantially higher in the WWTP samples than in surface water. This can be explained by dilution of the WWTP effluents in receiving surface water.

Cytotoxicity occurred often at lower REF than activation of AhR in surface waters and WWTP effluents (Figure 1A). Chemicals that are potent activators of AhR are often quite hydrophobic and therefore more bound to organic matter rather than freely dissolved, so are not captured by SPE. Therefore, it is not unexpected that many samples showed no activation of AhR up to the highest tested REF of 100. The highest SR_cytotoxicity_ in AhR CALUX was 22 for the WWTP effluent in Riesa. The tributary Mulde showed a SR_cytotoxicity_ of 14 for AhR, indicating that there might be more potent or higher concentrations of xenobiotic metabolism inducing chemicals. These SR_cytotoxicity_ aligned well with previous studies on the same water types (Figure S4).

Comparing the EC_IR1.5_ and IC_10_ for the AREc32 bioassay in Figure 1B, no sample was above a SR_cytotoxicity_ of 10, with many samples only cytotoxic and not activating the oxidative stress response. In contrast, the sampling sites Tangermünde (right), Wittenberge (middle) and Werben (left) showed relatively high SR_cytotoxicity_ of 9.9, 9.2 and 8.5, respectively, which is in accordance with their low EC_IR1.5_ values (Figure S5). Kamjunke et al.^36^ concluded that micropollutant concentrations from diffuse sources (e.g., pesticides) increased while those from point sources (e.g., pharmaceuticals) decreased along the river stretch. Therefore, the continuous discharge of e.g., pesticide metabolites in the Elbe River can increase with increasing discharge and the high effect of the AREc32 assay in the region around Tangermünde could be explained due to continuous runoff from the agricultural land upstream.

Regarding estrogenicity (Figure 1C), effects in the WWTP influents occurred at much lower REFs than for other end points and SR_cytotoxicity_ were higher than for other end points. The influent of the WWTP in Hetlingen showed the highest SR_cytotoxicity_ of 100 for estrogenicity. The WWTP in Meißen showed an increase in SR_cytotoxicity_ by a factor of 5 after treatment. The influent and effluent samples were taken approximately with the mean hydraulic retention time of the WWTPs, but the retention time in the WWTP of the 24-h composite samples (usually from 8:00 am to 8:00 am) could have been affected by, e.g., mixing and peak loads, resulting in non Lagrangian sampling conditions. For surface water, the sampling sites Lauenburg and Dömitz showed high SR_cytotoxicity_ values above 10, indicating specifically estrogenic chemicals. In Zehren no effect measurement of the water samples was possible at all three lateral sampling sites due to masking by cytotoxicity, presumably caused by a high input of the WWTP effluent in Meißen before Zehren (EC_10_ 2.28 REF). Comparing the SR_cytotoxicity_ with the results of the study from Lee et al.^12^ who had investigated small creeks in agricultural areas during rain events, Lauenburg right and Dömitz left are approximately in the same range as the highest SR_cytotoxicity_ of the German small creek samples (Figure S8). In contrast, Barrow et al.^37^ and Caracciolo et al.^38^ reported a lower average SR_cytotoxicity_ (2.62 and 2.24, respectively) compared with the German creeks and Elbe (3.48 and 3.70, respectively), indicating more specific acting chemicals in the Elbe.

For neurotoxicity (Figure 1D), the surface water Obristvi, the tributary Bilina, the surface water Neu Darchau right and tributary Jizeria had the lowest EC_10_ values of 1.27 ± 0.29 REF, 4.44 ± 0.03 REF, 5.30 ± 0.05 REF, 5.80 ± 0.45 REF, respectively (Figure S9). The highest SR_cytotoxicity_ (2.00–1.66) values were detected in Neu Darchau and Lauenburg and Torgau, suggesting an inflow of chemicals inhibiting neurite outgrowth. Comparatively, for samples with similar cytotoxicity, the shortening of neurites tended to have lower EC_10_ values in surface water than in WWTP effluents, with the exception of the effluent in Hetlingen. The Bilina River flows through an area with a history of extensive heavy industry, brown coal mining and associated chemical industries, which contributes to its petrochemical pollution.^39^ Additionally, there is significant municipal wastewater impact, indicated by high levels of contaminants such as sucralose, reflecting the influence of a highly populated area on this relatively small river. The higher sensitivity for neurite outgrowth in surface water samples compared to WWTP effluent could be attributed to the presence of agricultural pesticides or chemicals from road runoff, which are often released into water bodies during rain events. This observation aligns with the findings of Lee et al.,^14^ although they reported much higher SR_cytotoxicity_ in their samples (Figure S10). It is important to note that their sampling campaign focused specifically on rain events and agricultural runoff, which likely led to the detection of higher concentrations of specific chemicals affecting neurite outgrowth than the baseline monitoring conducted on the Elbe. SR_cytotoxicity_ of WWTP influents from Magdeburg, Riesa and Dresden were in the range of a survey on European WWTP effluents, but other influents and all effluents acted remarkably nonspecific (SR close to 1).

### Water Quality Assessment

Effect-based trigger values are important to differentiate between acceptable and poor surface water quality. They are not accepted in regulation yet, apart from recycled water quality in the State of California,^40^ but provide an important benchmark for research applications. The previously derived EBTs are 250 ng/L BaP-EQ for the AhR assay, 1.4 mg/L Dichlorvos-EQ for oxidative stress response and 0.34 ng/L Estradiol equivalent concentration (EEQ) for the ERα-GeneBLAzer assay.^3^ There does not exist an EBT for neurotoxicity but a method for derivation of interim EBTs has been recently proposed.^41^ The EBT Narciclasine-EQ for neurotoxicity derived with this method was 283 ng_Narciclasine_/L.

While all the B­[*a*]­P-EQ, Dichlorvos-EQ and Narciclasine-EQ for the Elbe surface water samples were below their respective EBT (Figure 2A,2B,2D), the EEQ exceed the EBT of 0.34 ng_EEQ_/L of Escher et al.^32^ in 14% of the surface water sampling sites (Figure 2C): Tidal Elbe 19 close to Geesthacht (1.07 ± < 0.01 ng_EEQ_/L), Riesa right (1.03 ± < 0.01 ng_EEQ_/L), Lauenburg right (0.96 ± < 0.01 ng_EEQ_/L) and left (0.80 ± < 0.01 ng_EEQ_/L), Schnackenburg middle (0.56 ± < 0.01 ng_EEQ_/L), Riesa left (0.56 ± < 0.01 ng_EEQ_/L), Dömitz left (0.52 ± < 0.01 ng_EEQ_/L), Riesa middle (0.44 ± < 0.01 ng_EEQ_/L), Lauenburg middle (0.40 ± < 0.01 ng_EEQ_/L), Tangermünde left (0.38 ± < 0.01 ng_EEQ_/L), Geesthacht right (0.37 ± < 0.01 ng_EEQ_/L), Lauenburg middle (0.35 ± < 0.01 ng_EEQ_/L). Comparison of these EEQ measured in the Elbe with existing literature data (Figure S8) revealed that surface water EEQ of the Elbe surface water exhibited smaller ranges as those reported by Lee et al.^12^ who analyzed 85 small stream samples collected during rain events that were impacted by agriculture, indicating the high dilution capacity of the Elbe.

**Figure 2:**
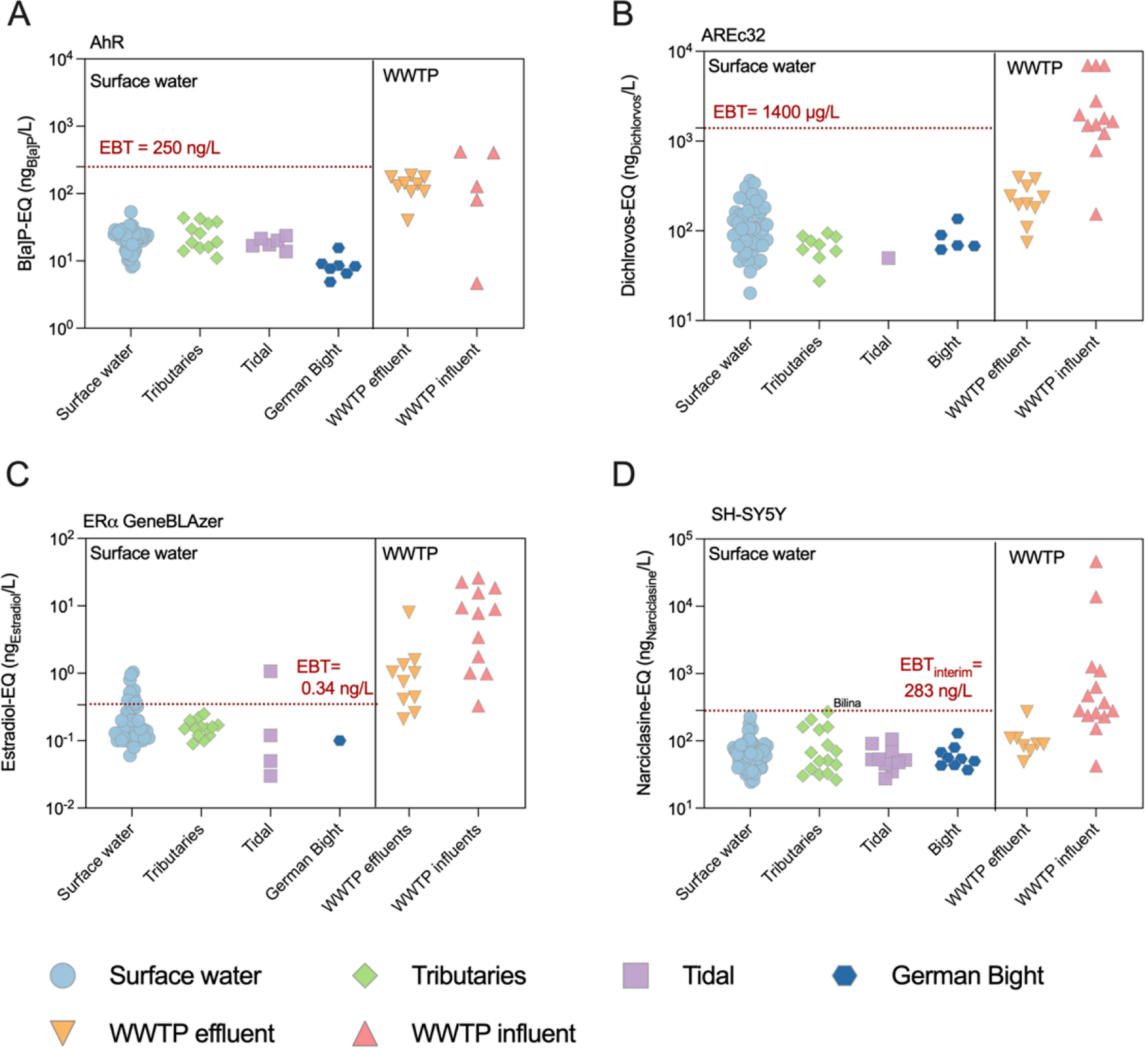
Comparison of bioanalytical equivalent concentration BEQ measured in the present study with literature data (add references) for (A) AhR CALUX; (B) AREC32, (C) ERα GeneBLAzer and (D) the neurotoxicity assay. Surface water blue circles, tributaries green diamonds, tidal violet squares, German bight dark blue circles, WWTP effluents orange downward facing triangle, WWTP influent red upward facing triangle.

Kidd et al.^42^ demonstrated that prolonged exposure of fathead minnows () to low concentrations (5–6 ng/L) of 17α-ethinylestradiol, a potent estrogen, led to the feminization of male fish and posed a substantial risk to the species inhabiting the lake under investigation. This underscores the potential hazard posed by estrogen and its analogs in freshwater ecosystems, which could compromise the survival of indigenous fish populations. Furthermore, Hecker et al.^43^ identified elevated levels of vitellogenin in fish sampled from the river Elbe, a common biomarker for estrogen exposure indicating a potential disruption of the endocrine system in these aquatic organisms. Subsequent studies have corroborated these findings, suggesting that parasitic infestation may also impact the endocrine system and reproductive capabilities of fish populations.^44^ These authors proposed that a combination of pollution and parasitism could synergistically affect the health and reproductive success of fish in the river Elbe.^44^ Given these findings and the observed exceedance of the EBT-EEQ of 0.34 ng/L in 14% of the surface water samples, the ecosystem of the Elbe may face significant threats due to endocrine disruption.

Könemann et al.^11^ demonstrated that measured EEQ_bio_ correlated well with EEQ_chem_ from analytical data and mixture models. This means that bioanalysis is sufficient to characterize estrogenicity in water samples, with the ratio of the very potent steroidal estrogens like the natural hormones estrone, 17β-estradiol and the synthetic hormone 17α-ethinylestradiol to less potent chemicals like bisphenols typically constant in surface waters. Effect-based methods can detect estrogenic substances at subng or even pg levels and have the potential to be used as complementary and reliable screening tools.

### (Mixture) Effect of Detected Chemicals

A total of 487 out of 713 analyzed chemicals (Table SE4) were detected in at least one water sample (Table SE5). The number of chemicals detected per site ranged from 39 (sample German Bight-15) to 265 (Bílina). The mixture effects of the detected and bioactive chemicals (Table SE6) were expressed as BEQ (Table S8). The Narciclasine-EQ_chem_ showed significant impact of the tributaries Bilina, Mulde and Saale and the surface water sample Obristvi to the load of neurotoxic chemicals to the Elbe River (Figure 3). The sampling sites with low EC_10_ (high neurotoxicity) are marked with blue boxes and are similar for the BEQ_chem_ for the Bilina and Obristvi site. Distinct spatial patterns in the distribution and concentration of various chemical categories along the Elbe River can be identified in the heatmap. The effect-scaled chemical profile (BEQ_chem_) varied along the river for each chemical category (Table SE7), with some sites dominated by polymer additives and others showing higher BEQ_chem_ of pharmaceuticals and personal care products. The categories intermediate, pharmaceuticals and pesticides occurred ubiquitously but contributed minimally to the samples’ BEQ_chem_, as shown by their generally lighter coloration across the map. Polymer additives, personal care products and additives were also ubiquitous but with higher contribution to the total BEQ_chem_. Dyes and biocides showed localized clusters, with biocides mostly found in first half of the Elbe and dyes clustered in the tidal samples. PFAS (per- and polyfluoroalkyl substances) showed some clustering in the middle section of the river with a small contribution to the total BEQ_chem_. While WWTP influents were dominated by biocides among those chemicals with effect data for the neurotoxicity assay, the WWTP effluents were dominated by pharmaceuticals and personal care products (Figure S11), which is consistent with previous work on neurotoxic chemicals in WWTP effluents where pharmaceuticals were the largest contributor to BEQ_chem_.^12^

**Figure 3:**
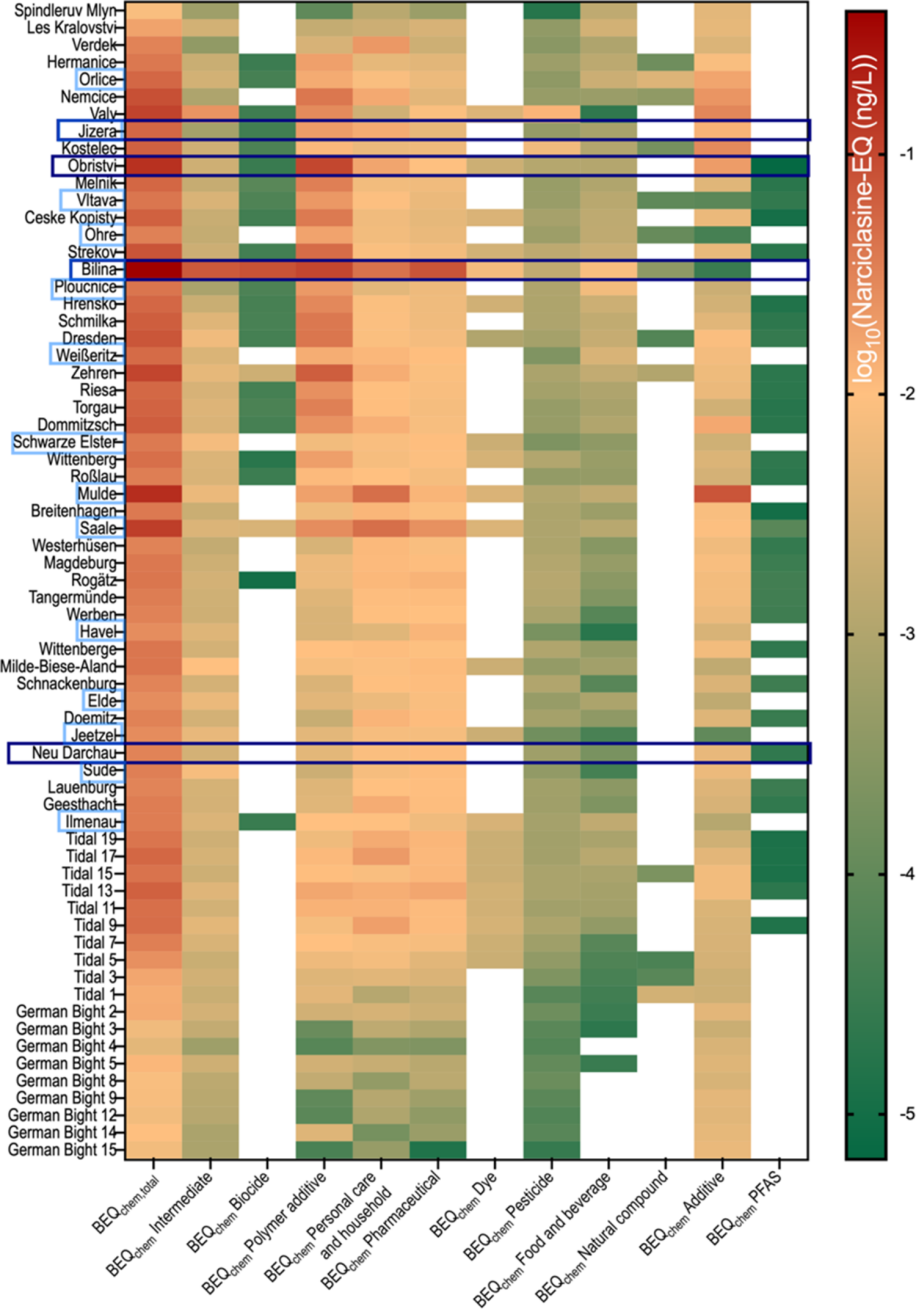
Categorical chemical profile BEQ_chem_ of surface water of the Elbe from the neurotoxicity assay. Contribution of chemical categorized to the predicted mixture effect Narciclasine-EQ for surface water sampling sites, tributaries are marked with light blue boxes, hotspots identified with bioassays are marked with dark blue boxes.

Typical markers of treated and untreated wastewater (WW) contamination, such as the pharmaceutical carbamazepine (treated WW), the artificial sweetener sucralose (untreated and treated WW) and caffeine (untreated WW), were detected and occurred at high concentrations at several sites. Notably, the Bilina River showed the highest concentration of caffeine (710 ng/L) and the third highest concentration of carbamazepine (following the tributary Saale and Tidal 13, with 98, 86, and 70 ng/L, respectively), indicating significant influence from untreated and treated wastewater. As caffeine has a low REP_*i*_, its contribution to the neurotoxicity mixture effect, i.e., its BEQ_caffeine_, remained small (food and beverage category in Figure 3).

Also evident from Figure 3 as well as for other end points is a decline of BEQ_chem_ in the Tidal section and further on in the German Bight due to dilution. In contrast such a decline was not observed for the BEQ_bio_ values (see Figure S12 as BEQ_chem_/BEQ_bio_ ratio). Thus, the contribution of known micropollutants, to the BEQ_bio_ decreases, suggesting that naturally occurring substances increase their share in contributing to effects in the bioassays.

There was high diversity in the chemicals contributing to the mixture effects, as Figure 4 demonstrates, and the number of chemicals contributing to the effect on the example of surface water (1–8 chemicals needed to explain 90% of explained effect, Table SE9). Shared mixture toxicity drivers for surface water which are under the top ten mixture toxicity contributors are 1-naphthol, 1,3 - diphenylguanidine, 2-benzothiazolesulfonic acid, 2-(methylthio)­benzothiazole, *N*-cyclohexyl-2-benzothiazole-sulfenamide. There was little commonality between sample types, and the major toxicity drivers varied significantly between the three bioassays for WWTP effluent compared to surface water (e.g., SH-SY5Y wastewater: 1,2-benzisothiazolinone, mebendazole, 1-naphthol; SH-SY5Y surface water: hexadecyltrimethylammonium, 1,3-diphenylguanidine and 2-(methylthio)­benzothiazole, Figure S13).

**Figure 4:**
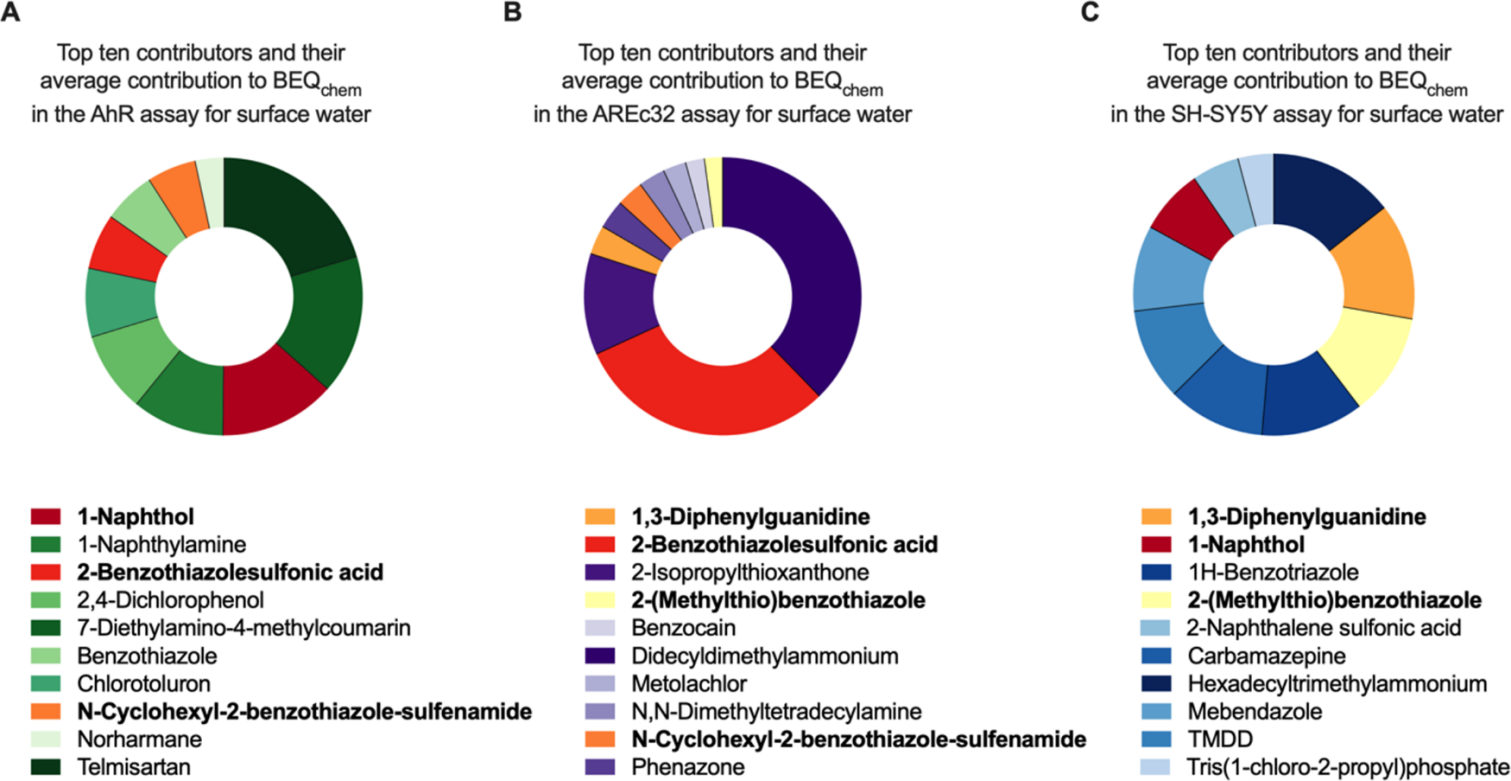
Top ten effect drivers of predicted mixture effects expressed as contribution of bioanalytical equivalent concentrations of detected chemicals (BEQ_i_) to the sum of the BEQ_i_ (BEQ_chem_) in Elbe River surface water for (A) AhR, (B) AREc32 and (C) SH-SY5Y. Shared mixture toxicity drivers are indicated with red to yellow color scale, while mixture toxicity drivers for the AhR are marked in green, for the AREc32 assay in purple and for SH-SY5Y assay in blue.

### Iceberg Modeling

The detected chemicals explained only a small fraction (on average 1.2%) of the measured effects. To predict the mixture effects of the measured concentrations, the EC_10_ values from single chemicals in the literature were used to calculate the respective REP_*i*_ (Table SE6). Contributions of individual chemicals to BEQ_chem_ were derived for each sample extract in the three bioassays, AREc32, AhR CALUX and neurotoxicity. For the AhR assay, EC values were available for 74 of the 487 chemicals detected in the Elbe samples, for the SH-SY5Y for 92 and for AREc32 for 90 chemicals. In Figure 5 the BEQ_bio_ and BEQ_chem_ are plotted against each other and the diagonals visualize the percentage of effect explained by the quantified bioactive chemicals.

**Figure 5:**
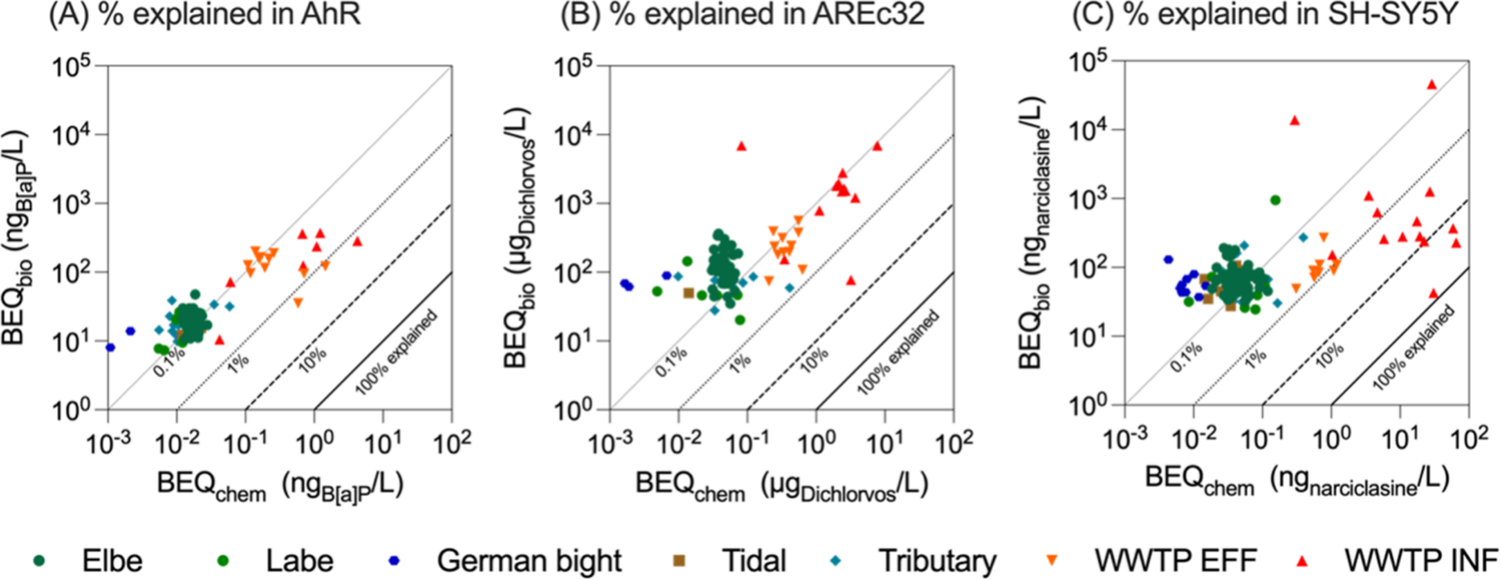
(A) Relationship between BEQ_bio_ and BEQ_chem_ (in ng/L or μg/L reference compound) for (A) the AhR-CALUX, (B) AREc32 and (C) neurite outgrowth inhibition on SH-SY5Y. Diagonal lines refer to % explained effect by detected chemicals.

For the AhR assay 0.0003–1.5% of the observed effect could be explained. The chemicals 7-diethylamino-4-methylcoumarin, 1-naphthol, and benzothiazole were among the top contributors to the AhR effect in all samples they were found (found in 26, 15, 11 samples, respectively). The chemical 7-diethylamino-4-methylcoumarin (a fluorescent dye) contributed 52% to BEQ_chem_ in the Pardubice WWTP effluent (0.58% of BEQ_bio_ explained), which treats wastewater from a chemical plant that produces pigments and dyes. 7-Diethylamino-4-methylcoumarin was identified as a highly potent antiandrogen and identified as a relevant environmental toxicant, which can be found at high concentrations at specific sites, like in the study of Muschket et al.^45^ The chemicals telmisartan, 2-benzothiazolesulfonic acid, 1*H*-benzotriazole were found in 127, 121, and 129 samples, respectively, and were the top contributor to BEQ_chem_ in 125, 102, and 89 samples, respectively.

The AhR is a ligand-dependent transcription factor for metabolic enzymes that is mainly activated by halogenated aromatic hydrocarbons, such as PAHs, PCBs and dioxins. These compounds are found in the environment, while very few of them (17 PCDD/Fs and 12 + 6 PCBs) are regulated. PAHs bind to suspended particulate matter and would not be expected in water samples filtered with a 0.7 μm filter. However, residual smaller particles and colloids may pass and be enriched by SPE, contributing to the unknown fraction of B­[*a*]­P-EQ_bio_. The 2016 ELSA report indicates that low-chlorinated PCBs originate from mining regions where they were used in hydraulic oils for underground ore extraction.^46^ These PCBs are emitted into the rivers Mulde and Saale through remaining drainage galleries and subsequently flow into the Elbe. While these PCBs are likely to remain largely bound to sediments, they are partially remobilized, contributing to high AhR activity.

In the study by Kamjunke et al.^36^ target screening of organic micropollutants revealed peaks of phosphate flame retardants (1.4 μg/L) at the Mulde estuary during extreme drought conditions, followed by the Saale estuary (807 ng/L). These findings are consistent with the low EC_10_ values observed for the Mulde and Saale estuaries in this study (Figure S3) and high concentrations found in the target screening (flame retardant 460, 310 ng/L Saale), which indicate the ongoing input of AhR-activating chemicals. Additionally, the Middle Elbe (from Wittenberg to Wittenberge) is considered a significant buffer and secondary source of persistent contaminants, such as PCBs.^46^ Temporary or permanent still water areas, such as oxbows, lakes, backwaters and groin fields, change their primary function from acting as a sink for micropollutants during low to medium headwater discharge to becoming a source during floods.^47^ Elevated PCB levels, notably up to the area of the lower Middle Elbe at Schnackenburg were measured by Schwartz et al.,^46^ consistent with measured EC_10_ values which are lowest in surface water for Schnackenburg, Neu Darchau and Dömitz, which are consecutive sampling points. In contrary, the SR_cytotoxicity_ values for Schnackenburg, Neu Darchau and Dömitz showed moderate specificity with SR_cytotoxicity_ from 3.4 to 5.8.

For oxidative stress, 10 chemicals explained 3.85% of the observed effect in the AREc32 assay in the municipal influent of the WWTP in Pardubice. These chemicals came from various categories: polymer additive, additive, food and beverage, intermediate, and biocide (e.g., *N*-isopropyl-*N*-phenyl-*p*-phenylenediamine, *N*,*N*-dimethyltetradecylamine, daidzein, 1-naphthol, genistein, chlorophene, 1-naphthylamine, 2-benzothiazolesulfonic acid). Dichlorvos-EQ_chem_ explained 0.69% of Dichlorvos-EQ_bio_ in the Bilina River (based on didecyldimethylammonium, 2-benzothiazolesulfonic acid, 1,3-diphenylguanidine, *N*,*N*-dimethyltetradecylamine, bisphenol A), followed by 0.38% of BEQ_bio_ at the Obristvi site (based on 2-benzothiazolesulfonic acid, 1,3-diphenylguanidine, benzocaine, phenazone, metolachlor). According to Lee et al.,^12^ the industrial chemical 2-benzothiazolesulfonic acid, which is often detected in WWTP effluent samples, was characterized as a main toxicity driver for the oxidative stress response. In this study, 2-benzothiazolesulfonic acid was the top contributor for oxidative stress in 119 samples. In previous studies, only a small fraction of the sample’s effect in assays indicative of xenobiotic metabolism and adaptive stress responses could be explained by the quantified chemicals.^7^ This is likely due to the thousands of nonquantified chemicals expected to be present in water samples that contribute to effects.

For neurite outgrowth inhibition, with few exceptions, less than 0.07% (median, average 1.26%) of the measured mixture effects were explained by the predicted mixture effects of the detected chemicals for surface water. Overall, Narciclasine-EQ_bio_ was better explained by Narciclasine-EQ_chem_ in WWTP influents than in surface water (median % explained 0.08, average 3.43%). 1,2-Benzisothiazolinone had an average contribution of 62% of the total Narciclasine-EQ_chem_, followed by the very potent neurotoxicant mebendazole (REP 0.0367) with 11% and 1*H*-benzotriazole with 11%. Narciclasine-EQ_chem_ could explain 72% of Narciclasine-EQ_bio_ in the industrial influent in Pardubice, with chemicals 1-naphthol (10 μg/L), 1,2-benzisothiazolinone (0.24 μg/L), 2,4-dinitrophenol (24 μg/L) contributing 90% to Narciclasine-EQ_chem_. The tributary Mulde and the surface water Strekov could be explained to 0.55 and 0.32%, respectively.

## Conclusions

Despite the analytical quantification of a substantial number of chemicals (487 out of 713 targeted), iceberg modeling revealed that this number is insufficient to fully account for the observed mixture effects in environmental waters. Nontarget analysis can help to identify unknown chemicals but for risk assessment the quantities need to be known, too, although there are novel machine-leaning approaches to prioritize features identified in nontarget screening by their anticipated hazard.^48^ Expanding the testing to include even more chemicals may not resolve this issue as it would necessitate the analysis of hundreds to thousands of additional compounds and the characterization of all detected chemicals in the *in vitro* bioassay test battery. Furthermore, we have evidence from earlier studies that chemicals below their detection limit might still contribute to the mixture effects in a concentration-additive manner.^49^ Designed mixture studies with chemicals from surface water at concentration ratios they were detected have provided evidence that synergistic effects are unlikely to be the cause of the underestimation of the mixture effects because all designed mixtures acted according to the mixture concept of concentration addition.^7,50^ Additionally, the dissolved organic matter (DOM) content can complicate the detection and characterization of chemicals in water samples. Moreover, chemicals present below analytical detection limits, along with a vast array of degradation products and unknown substances, can contribute significantly to mixture effects. To address these challenges comprehensively, we recommend the combined use of iceberg modeling to capture the full extent of mixture effects and understand the relative contributons of known environmental pollutants, especially for estrogenic chemicals, which occur at low concentrations, at which analytical quantification often remains below the method detection limit, while bioassays are more sensitive for this effect class. Effect-directed analysis (EDA)^51^ is suggested as a valuable addition for identifying causative chemicals for specific MoAs, like estrogenicity, to identify main toxicity drivers. Additionally, creating artificial mixtures can enhance our understanding of the chemicals driving observed effects. This includes developing EBTs, such as those derived here for the SH-SY5Y assay, which can effectively differentiate between acceptable and concerning water quality.

## Supplementary Material





## Data Availability

Data availability statement. All data tables are are compiled and referenced in the Supporting Information, the bioassay results with plots of all samples are deposited in Zenodo (https://zenodo.org/doi/10.5281/zenodo.12806297).

## Notes

Additional information on sampling sites, sample extraction, LC-HRMS analysis of sample extracts, experimental methods of the bioassays, additional results (PDF)
The sampling information (SE = supporting data in excel sheet, Table SE1), quality control data of the bioassays (Table SE2), experimental data of the bioassays (Table SE3) data of chemical analysis (Table SE4 and SE5) and single chemical effects (Table SE6) used for the iceberg modeling as well as the results of the iceberg model (Tables SE7–SE9) (XLSX)

## Abbreviations

AhR: aryl hydrocarbon receptor
B­[*a*]­P: benzo­[*a*]­pyrene
BEQ: bioanalytical equivalent concentration
CRC: concentration response curve
CV: coefficient of variation
DOM: dissolved organic matter
EBT: effect based trigger value
ECIR1.5: effect concentration causing an induction ratio (IR) of 1.5
EC10: 10% effect concentration
EDA: effect-directed analysis
EEQ: estradiol equivalent concentration
ER: estrogen receptor
HTS: high throughput screening
IC10: 10% inhibition concentration
IR: induction ratio
LC-HRMS: liquid chromatography high-resolution mass spectrometry
MoA: mode of action
REF: relative extraction factor
REP: relative effect potency
RLU: relative light units
SE: standard error
SR: specificity ratio
SPE: solid phase extraction
tBHQ: *tert*-butylhydroquinone
TCDD: 2,3,7,8-tetrachlorodibenzodioxin
WWTP: wastewater treatment plant
