# Supplementary material for: Following the Mixtures of Organic Micropollutants with In Vitro Bioassays in a Large Lowland River from Source to Sea
Source: ACS Environ Au. 2025 Jan 19;5(4):363–75. doi: 10.1021/acsenvironau.4c00059 (PMC12272280; doi:10.1021/acsenvironau.4c00059)
Supplement: Supplementary file 1 [file vg4c00059_si_001.pdf]

Supporting Information

Following the mixtures of organic micropollutants with *in vitro* bioassays in a large lowland river from source to sea

Elena Hommel[a], Maria König[a], Georg Braun[a], Martin Krauss[b], Norbert Kamjunke[c], Werner Brack[b,d], Anna Matousu[e], Tina Sanders[f], Ingeborg Bussmann[g], Eric P. Achterberg[h], Björn Raupers[h], Beate I. Escher[a,i*]

[a]Department of Cell Toxicology, Helmholtz Centre for Environmental Research – UFZ, Permoserstr. 15, Leipzig 04318, Germany

[b]Department Exposure Science, Helmholtz Centre for Environmental Research – UFZ, Permoserstr. 15, Leipzig 04318, Germany

[c]Department of River Ecology, Helmholtz Centre for Environmental Research - UFZ, Brückstr. 3a, Magdeburg 39114, Germany

[d]Institute of Ecology, Evolution and Diversity - Goethe University, Max-von-Laue-Str. 13, Frankfurt am Main 60438, Germany

[e]Biology Centre, Czech Academy of Sciences, Institute of Hydrobiology, Ceské Budejovice 370 05, Czech Republic

[f]Institute of Carbon Cycles, Helmholtz Centre Hereon, Max-Planck-Straße1, Geesthacht 21502, Germany

[g]Department of Shelf Sea System Ecology, Alfred-Wegener-Institut, Helmholtz Zentrum für Polar- und Meeresforschung, Kurpromenade 201, Helgoland 27498, Germany

[h]GEOMAR, Helmholtz Centre for Ocean Research Kiel, Wischhofstraße 1-3, Kiel 24148, Germany

[i]Environmental Toxicology, Department of Geosciences, Eberhard Karls University Tübingen, Schnarrenbergerstr. 94-96, Tübingen 72076, Germany

Table of Contents

Text S1. Sampling sites 5

Text S2. Sample extraction 7

Text S3. LC-HRMS analysis of sample extracts 9

Text S4. Experimental methods of the bioassays 11

Text S5. Additional Results 16

List of Tables

Table S1. Overview of sampling sites in different Elbe regions and their special features. 5

Table S2. Method for solid phase extraction of organic micropollutants on a Promochrom SPE-03 device. 8

Table S3. Composition of AhR CALUX and AREc32 cell media. 11

Table S4 Composition of ERα-GeneBLAzer™ cell media. 11

Table S5. Composition of the SH-SY5Y cell media. 12

Table S6. Test battery of bioassays and corresponding reference compounds used, with concentration range in M in the bioassay, provider and literature reference. 15

List of Tables in the separate Excel SI

Table SE1. Sampling sites, with coordinates, Elbe kilometer, sampling and extraction day ordered from low to high Elbe kilometer.

Table SE2. Effect concentrations of reference chemicals in SH-SY5Y, AhR-CALUX, ERα GeneBLAzer, and AREc32. Reported are mean EC from individual EC_{10} and IC_{10} values below. Sample replicate EC_{10} values in the four bioassays are below.

Table SE3. Inhibitory and effect concentrations for surface water and WWTP effluent in SH-SY5Y, AhR-CALUX, AREc32, and ERα-GenBLAzer (REF: relative enrichment factor, $L_{water}/L_{bioassay}$). For all calculations the linear model was used, if log-logistic model is used it is indicated in the table. results generated with the automated bioassays screening in R.

Table SE4. Compound information of detected concentrations and tested chemicals not detected in the Elbe.

Table SE5. Concentrations (ng/L) of the 487 detected target analytes in the Elbe samples and 226 non detected chemicals.

Table SE6. Available EC_{10} values for measured chemicals for the SHSY5Y, AhR, AREc32 and ERa GeneBLAzer with respective reference.

Table SE7. Percent contribution of chemical category to respective reference-compound EQ_{chem} (e.g., sum of dichlorvos-EQ_i of n chemicals i of the same category divided by dichlorvos-EQ_{chem}, analogous to eq. 11) in AREc32 for each Elbe water extract. Categories without contribution to dichlorvos-EQ_{chem} in any of the samples were not shown.

Table SE8. BEQ_{bio} and BEQ_{chem} [M of reference compound] for all samples in the AhR, AREc32 and SHSY5Y assay.

Table SE9: Number, name and category of chemicals that explain 90% of BEQ_{chem} and percent explained of $BEQ_{unknown}$.

List of Figures

Figure S1: Sampling locations along the Elbe River... 6
Figure S2. Sample replicates measure in the four bioassays. 16
Figure S3. The logarithmic values of the EC_{10} (effect concentration 10% in Relative extraction factor) for AhR assay from Spindleruv Mlyn to the German Bight are represented graphically, with smaller circles denoting wastewater treatment plant (WWTP) influents and effluents. 17
Figure S4. Comparison of effect data with literature.. 18
Figure S5. Oxidative stress response (AREc32) along the Elbe. 19
Figure S6. Comparison of oxidative stress response (AREc32) of the Elbe samples with literature. 20
Figure S7. logarithmic values of the EC_{10} (effect concentration 10% in REF (relative extraction factor)) for the ERα assay from Spindleruv Mlyn to the German Bight are represented graphically, with smaller circles denoting wastewater treatment plant (WWTP) influents and effluents. 21

Figure S8. Comparison of EEQ of the Elbe with literature. 22
Figure S9. Neurotoxicity (SH-SY5Y) along the Elbe. 23
Figure S10. Comparison of neurotoxicity (SH-SY5Y) of Elbe samples with literature data. 24
Figure S11. Categorical chemical profile BEQ_{chem} of wastewater treatment plant (WWTP) influent and effluents into the Elbe from the neurotoxicity assay. Contribution of chemical categories to the predicted mixture effect $BEQ_{chem,total}$ in Narciclasine- EQ. 25
Figure 12 BEQ_{chem} to BEQ_{bio} ratio from Wittenberge to the German Bight, indicating a decrease after sampling point tidal 9. 26
Figure S13. Toxicity driver for WWTP effluents and influents for the AhR, SHSY5Y and AREc32 assay. 27

Text S1. Sampling sites

Sample type, sampling coordinates, and sampling day can be found in Table S1. A short description of sampling sites is given in Table S1 and Figure S1.

Table S1. Overview of sampling sites in different Elbe regions and their special features.

Region	Number of sampling sites	Special features
Labe	12	No Langrangian sampling possible, as a stretch of 200 km is intersected by 25 weirs
Czech tributaries	6 (+ 1 replicate)	Replicate Vltava
WWTP influents Czech Republic	3	Pardubice influent municipal and industrial;
WWTP effluents Czech Republic	3	Neratovice influent: polishing pond from industrial WWTP
German part of the Elbe	20 x 3; (Dresden and Breitenhagen just 2, + 1 replicate Neu Darchau right), total 59	Lateral sampling Schmilka, Roßlau, different lateral sampling height
German tributaries	10 (+ 1 replicate)	Replicate Saale
Tidal Elbe	10	starting near Cuxhaven
German Bight	9	
WWTP influents Germany	10	Wittenberg industrial + municipal influent separately
WWTP effluents Germany	9	

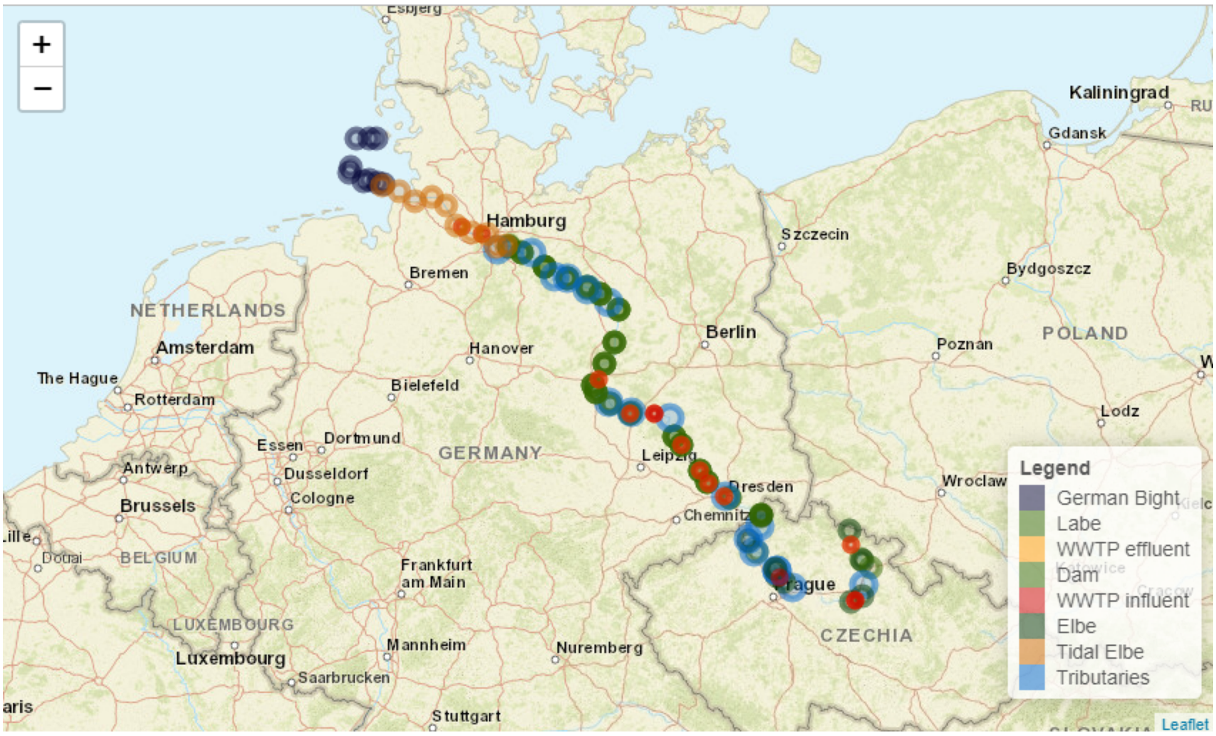

Figure S1: Sampling locations along the Elbe River.

The different sampling types are indicated with color. For the Czech part of the Elbe called Labe light green, for tributaries light blue, for the German Elbe dark green, for the tidal Elbe brown for the German Bight dark blue. WWTP influents and effluent are plotted on top of each other and are red.

Text S2. Sample extraction

The samples were received frozen and stored at -20°C. For the bioassays and chemical analysis, approximately 650 mL of the sample was thawed and vacuum filtered to remove suspended solids using glass fiber filters (Whatman GF/F, nominal 0.7 μm pore size) in solvent-rinsed glassware. After solid-phase extraction, the extracts were evaporated under a gentle nitrogen stream using a XcelVap® Automated Evaporation and Concentration System to approximately 1 mL. Afterwards the extracts were filtered with a syringe filter (PVDF, 0.2 μm, Phenomemex) while transferring into HPLC vials and were allowed to dry completely. Then the extracts were solubilized in MeOH to achieve an extraction factor (EF; $L_{water}/L_{extract}$, see equation S1) of 1000 for all samples (e.g. 650 μL of MeOH for 650 mL of samples, to yield an EF of 1000) and stored at -20°C. Field and processing blanks using 150 mL of LC-grade water were also filtered and underwent the same process.

$$EF=\frac{\text{Volume extracted}}{\text{final Volume extract}}\left[\frac{L_{water}}{L_{bioassay}}\right] \quad (S1)$$

Table S2. Method for solid phase extraction of organic micropollutants on a Promochrom SPE-03 device.

**Action**	**Inlet 1**	**Flow rate (mL/min)**	**Volume (mL)**
Elute W2	Ethyl acetate, LC-MS grade	20	5.0
Elute W2	Methanol, LC-MS grade	20	5.0
Elute W1	Water, LC-MS grade	20	5.0
Add Sample W1	Sample	10	900.0
Air-Purge W1	Air	20	10.0
Blow N2		Time based	120 min
Collect 2	Ethyl acetate, LC-MS grad	5	5.0
Air- Purge 2	Air	20	10.0
Collect 2	Methanol, LC-MS grade	5	5.0
Air- Purge 2	Air	20	10.0
Collect 2	Methanol + 1% of formic acid	5	5.0
Air- Purge 2	Air	20	10.0
Collect 2	Methanol + 2 % 7 N Ammonia in methanol	5	5.0
Air- Purge 2	Air	20	10.0

Methanol and ethyl acetate (both LC-MS grade) and 7 N ammonia in methanol (anhydrous) were purchased from Sigma-Aldrich, LC-MS-grade water was obtained from Fisher Scientific and formic acid (98 – 100 %; for analysis) was ordered from Merck.

Text S3. LC-HRMS analysis of sample extracts

A 50 µL aliquot of the extracts (EF 1000) was transferred into a 2 mL autosampler vial with insert, 15 µL of methanol, 30 µL of water and 5 µL of an internal standard mixture (1 µg/mL) containing 49 isotope labelled compounds were added (i.e., EF for analysis = 500). Instrumental blanks were prepared using methanol and water in a ratio of (70:30). WWTP influent and effluent samples were analysed at an EF of 50 to account for higher concentrations.

Method-matched calibration standards were prepared by spiking a mixture of the target analytes into 1 L of water from a pristine stream (Wormsgraben) from the upper Harz Mountains, Northern Germany. Twelve calibration levels were prepared ranging from 0.2 to 1000 ng $L^{-1}$and subjected to the same solid-phase extraction procedure as the samples.

The samples were analysed by an Ultimate 3000 LC system (Thermo), consisting of a ternary pump, autosampler and column oven, which was coupled to a quadrupole orbitrap instrument (Thermo Exploris 480) via a heated electrospray ionisation source (ESI). Separate runs were used for positive and negative ion mode. For chromatography, we used a Phenomenex Kinetex Biphenyl column (100 Å pore size; 100 x 2.1 mm; 2.6 µm particle size; with pre-column 5 x 2.1 mm and 0.2 µm in-line filter) at 40°C. For positive mode analysis, elution was done with a ternary gradient of 0.1% v/v formic acid (Eluent A) and methanol with 0,1% v/v formic acid (Eluent B) and acetonitrile (Eluent C) at a flow rate of 300 µL/min. In negative mode, instead of formic acid, 1 mM ammonium fluoride was used as eluent modifier. The gradient started A at 97% A / 3% B / 0% C, held for 2 min. Afterwards the fraction of B was linearly increased to 97% within 14 min and subsequently the fraction of C was increased to 97% C within 4 minutes, which were kept for 4 min. Finally, the column was re-equilibrated to the initial conditions for 5 min. The HRMS analysis used a combination of full scan acquisition (m/Z 80–1200) at a nominal resolving power of 60,000 (referenced to m/z = 200) and data-independent MS/MS (DIA) experiments at a nominal resolving power of 45,000. For the latter, the data were recorded using six broad isolation windows of the precursor ions (i.e., m/z ranges 80–182, 180–282, 280–382, 380–482, 480-682, 680-1200). Quantification was done using the full scan data, the DIA scans were used for the validation of the identity via one or two diagnostic fragments.

The Thermo .raw files were converted into the mzML format using ProteoWizard (v. 3.0.18265).[1] The software MZmine 2.38,[2] was subsequently used for peak detection and annotation of the target compounds as described in Pluskal et al.[3]. The R package MZquant[4] was used for the semi-automatic quantification of the analyte concentrations. Analytes showing broad peaks or high background levels difficult to detect and quantify by the semi-automated workflow were quantified using TraceFinder 5.1 (Thermo).

Method detection limits were determined based on the approach of the USEPA[5] from replicate injections (n=5) of the method-matched calibration standards.

Text S4. Experimental methods of the bioassays

Cell cultivation

The cell media streptomycin for AhR-CALUX and AREc32 cells were 90 % plus 10 % FBS, 100 µg/mL penicillin- streptomycin and 1 mg/mL Geneticin. For the ERα GeneBLAzer cell line 98 % Opti-MEM supplemented with 2 % charcoal-stripped FBS penicillin and 25mM HEPES, 0.1 mM NEAA, 0.1 mM Sodium Pyruvate and 100 µg/mL streptomycin. For SH-SY5Y to the 30 μL medium containing 97% Neurobasal medium with phenol-red, 2% B27 supplement, 1% GlutaMAX, 10 μM all-trans retinoic acid and 100 µg/mL penicillin- streptomycin. To the differentiated SH-SY5Y cells, 10 μM all-trans retinoic acid was added into each well containing 30μL medium. The samples and references were prepared in 1.5 mL conical short thread vials. To eliminate solvent effects, all methanolic water extracts were evaporated to dryness and reconstituted in assay media before bioanalysis. The application of the sample to the cells and subsequent serial dilution was performed using an automated liquid handling robot (Hamilton MICROLAB Star). The cells were then incubated for 24 hours at 37°C and 5 % CO_2.

Cell culture media

Table S3. Composition of AhR CALUX and AREc32 cell media.

Component	Concentration
DMEM with GlutaMAX (31966-021)	90%
Geneticin	1 mg/mL
Penicillin-Streptomycin	100 U/L, 100 μg/L
Untreated FBS	10%

Table S4 Composition of ERα-GeneBLAzer™ cell media.

Component	Concentration
dFBS	2%
HEPES	25 mM
NEAA	0.1 mM

Penicillin-Streptomycin	100 U/mL, 100 μg/L
Phenol red free DMEM with GlutaMAX	90%
Sodium Pyruvate	1mM

Table S5. Composition of the SH-SY5Y cell media.

Component	Concentration
all-trans retinoic acid	10 μM
B27 supplement	2%
GlutaMAX	2 mM (1%)
Neurobasal medium with phenol-red	97%
Penicillin-Streptomycin	100 U/mL, 100 μg/L

Measuring of cytotoxicity

The cell viability and cytotoxicity were determined by measuring and comparing the confluency of the exposed and unexposed cells using equation S2:

$$\%\ \text{Inhibition of cell viability} = 100\% - \frac{\%\ \text{confluency (exposed cells/sample)}}{\%\ \text{confluency (unexposed cells)}} \qquad \text{(S2)}$$

Detection of activation in AREc32 and AhR CALUX

After 24hours of exposure and measurement of cell confluency, AREc32 and AhR CALUX cells were washed twice with 150 μL PBS. Subsequently, 10 μL of lysis buffer containing 25 mM Tris (AppliChem, A13790500), 1 % Triton-X100 (GeyerChemsolute, 8059), 2 mM EDTA (AppliChem, A11040500), 2 mM DTT (Sigma-Aldrich, D0632), 10% glycerol (AppliChem, A11231000) was added to each well, followed by a brief centrifugation (20 s at 100 rpm). The samples were then incubated for 15-20 minutes at room temperature and shaken to ensure complete cell lysis. Following this, 40 μL of luciferase substrate buffer (pH adjusted to 7.7-7.8) were added, containing 20 mM Tricine (Sigma-Aldrich, T0322), 2.67 mM $MgSO_4$ (AppliChem, 131404.1210), 33.3 mM DTT (Sigma-Aldrich, D0632), 0.1 mM EDTA (AppliChem, A11040500), 0.261 mM coenzyme A (Sigma-Aldrich, C3144), 0.53 mM ATP (Sigma-Aldrich A2383),

0.235 mM D-luciferin (AREc32) and 0.059 mM D-luciferin (AhR) (AAT Bioqest, ABD-12506). Finally, the luminescence was measured with a TECAN Infinite® M1000 plate reader (TECAN Trading AG, Männedorf, Switzerland).

Detection of Estrogen receptor activation

The detection of activation in the GeneBLAzer® assays encoding for the β-lactamase was earlier described by König et al.[6]. For the detection of the expression of β-lactamase the ToxBLAzer detection reagent was prepared according to the instructions of the manufacturer (ThermoFisher Scientific) and 8 µL of the reagent were added per well.[6] This substrate easily enters the cell, where endogenous esterases quickly convert it into a fluorescent precursor (blue fluorescence) which is then transformed into an enzyme product by β-lactamase (green fluorescence). The fluorescence was read using the Tecan Infinite® M1000 plate reader (TECAN Trading AG, Männedorf, Switzerland) with excitation at 409 nm and emission at 460 nm (blue) and 530 nm (green). As the ToxBlazer™ allows radiometric detection at these wavelengths, immediately after addition of the reagent (t = 0h) and after 2 hours of incubation at room temperature in the dark. The expression of the reporter gene β-lactamase was quantified using a blue/green screening method, using the following equation S3:

$$\frac{B}{G}=\frac{\left(E_{460\,nm}(2h)-\left(E_{460nm}(0h)-E_{460\,nm}(0h,\text{ unexposed cells})\right)\right)-E_{460\,nm}(2h,\text{ cell free})}{\left(E_{530\,nm}(2h)-\left(E_{539nm}(0h)-E_{530\,nm}(0h,\text{ unexposed cells})\right)\right)-E_{530\,nm}(2h,\text{ cell free})} \quad \text{(S3)}$$

The maximum B/G was determined using the concentration-response curve (CRC) of the reference compound 17-ß-Estradiol (E2) and the effect of the samples was calculated using the following equation S4:

$$\%\text{ effect}=\frac{\frac{B}{G}\text{ratio (sample)}-\frac{B}{G}\text{ratio (unexposed cells)}}{\frac{B}{G}\text{ratio (maximum)}-\frac{B}{G}\text{ratio (unexposed cells)}} \quad \text{(S4)}$$

Detection of neurite outgrowth

Neurite length was determined using an IncuCyte® S3 live cell imaging system using an established image analysis workflow from Lee et al., 2022a. Phase-contrast images were acquired at 10x magnification after 24 h exposure, covering 36 % of each well. Neurite outgrowth was measured by comparing neurite length with unexposed cells used as a control. Cell viability was assessed by live cell staining with Nuclear Green ® LCS1 (10 μM in PBS) and propidium iodide (1 μM in PBS). After acquisition of phase contrast images, cells were stained with 10 μL of staining solution and then incubated for 1.5 h. Stained cells were counted using the Basic analyzer mode of the IncuCyte® S3 software. Fluorescence images were captured using a 10x zoom objective in green (excitation wavelength: 460 nm; emission wavelength: 524 nm; exposure time: 300 ms) and red (585 nm; 635 nm; 400 ms) fluorescence channels. Cell viability was calculated by dividing the number of live cells (total dead cells) by the total number of cells using the Basic analyser mode of the IncuCyte® S3 software. Cytotoxicity was defined as the decrease in cell viability compared to unexposed cells.[7]

Blank correction

Thirteen processing blanks, one laboratory blank and 10 field blanks were prepared for the bioassays to estimate the impact of sampling and processing of the samples. Values exceeding a confidence threshold of 0.7 were considered as positive blanks and a blank correction procedure was applied to the samples extracted on the same day as these positive blanks. During our analysis, positive effects were observed for the AREc32 processing blank prepared on first and third of August 2023. Additionally, a response was noted for ERα on the samples collected in 26 July 2023 and 2 August 2023. To maintain data integrity, the equation S5 was used for blank correction. The condition that the Effect Unit (EU) of the processing blank remained below 50% of the EU exhibited by the sample was given for all four cases.

$$EU_{blank\ corrected} = EU_{sample} - EU_{blank} \quad (S5)$$

$$\frac{1}{EC10_{blank\ corrected}} = \frac{1}{EC10_{sample}} - \frac{1}{EC10_{blank}}$$

These blank effects can be caused by impurities of the solvents and leaching from materials used during the enrichment process.

No cytotoxicity was detected at any tested REF in the blanks, except for the field blank Neu Darchau with an IC_{10} of 23.35 ± 0.16 REF. While no cytotoxicity was observed in the respective processing blank, the samples Neu Darchau left, middle and right showed cytotoxicity and were therefore corrected as shown in equation 16 but with the IC_{10} instead of the EC_{10}. This measure helped to maintain the accuracy and reliability of our findings while mitigating any potential biases introduced by processing artifacts.

Reference compounds

Table S6. Test battery of bioassays and corresponding reference compounds used, with concentration range in M in the bioassay, provider and literature reference.

Bioassay	Reference compound (concentration range)	Purchased from	Reference
AhR CALUX	TCDD<br>$2.13 \cdot 10^{-10}$ M – $2.08 \cdot 10^{-14}$ M	Dr. Ehrenstorfer Standards (Germany)	Brennan et al. [8]
AREc32	tBHQ<br>$1.73 \cdot 10^{-5}$ M – $1.35 \cdot 10^{-8}$ M	Sigma Aldrich (Castle Hill, Australia).	Wang et al.[9]<br>Escher et al.[10]
ERα-GeneBlazer	17-ß-Estradiol<br>$1.38 \cdot 10^{-8}$ M – $1.31 \cdot 10^{-13}$ M	Sigma Aldrich (Steinheim, Germany)	König et al.[6]
SH-SY5Y	Narciclasine<br>$1.87 \cdot 10^{-5}$ M – $9.13 \cdot 10^{-11}$ M	Sigma Aldrich (Steinheim, Germany)	Lee et al.[7]

Text S5. Additional Results

Quality control

Three replicates were taken for the Vltava, Saale and Neu Darchau right side sampling sites (figure 2). While the EC_{10} results for the AhR and AREc32 assays are very close, some variance was observed for the SHSY5Y assay at Vltava. Also, for the ER GeneBLAzer assay, only one sample was not masked by cytotoxicity for the Vltava and Saale sampling sites. However, the Neu Darchau replicate also showed close results. In conclusion, the bioassays provide a robust result.

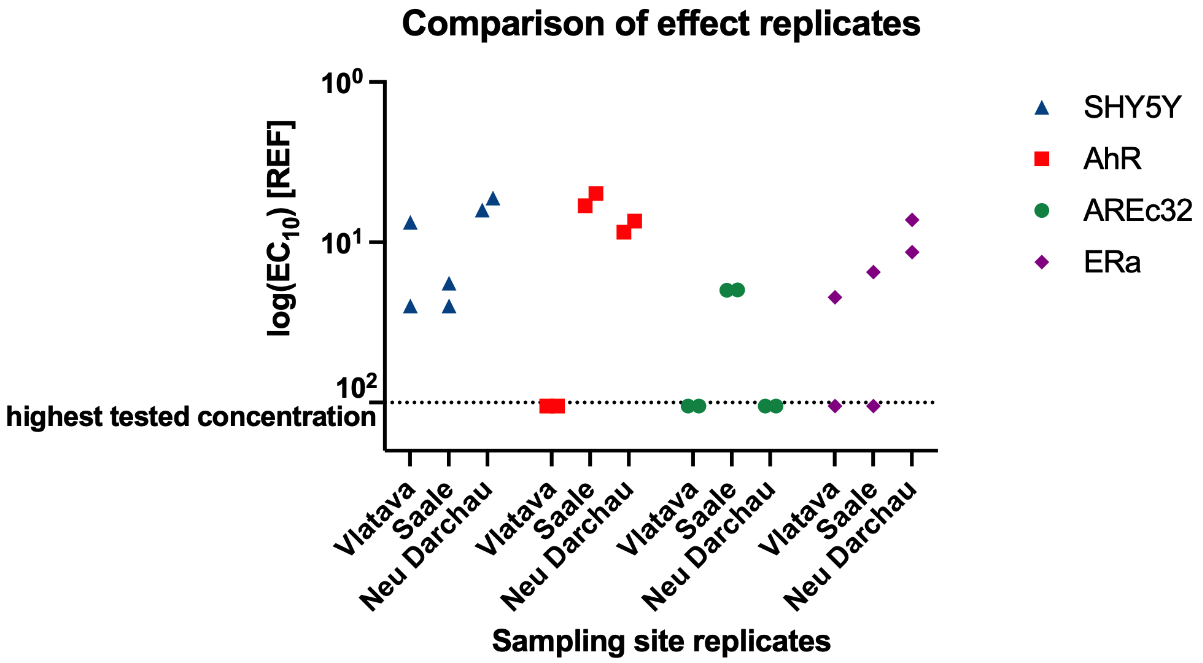

Figure S2. Sample replicates measure in the four bioassays.

Frequency of detected effects and cytotoxicity

Table S7. Frequency of EC_{10} and IC_{10} detected of ERα, AhR, AREc32, SHSY5Y bioassays for a total of 133 samples.

	AhR	ERα	AREc32	SHSY5Y
EC high confidence/ low confidence	100/0	87/0	87/0	116/11
EC>IC / no effect but cytotoxicity	0/31	0/40	5/39	4/0
No effect and no cytotoxicity	2	6	2	2

IC high confidence/ low confidence	65/48	10/93	40/61	51/55
No cytotoxicity (<100 REF)	20	30	32	27

Hotspots of the aryl hydrocarbon receptor mediated activity

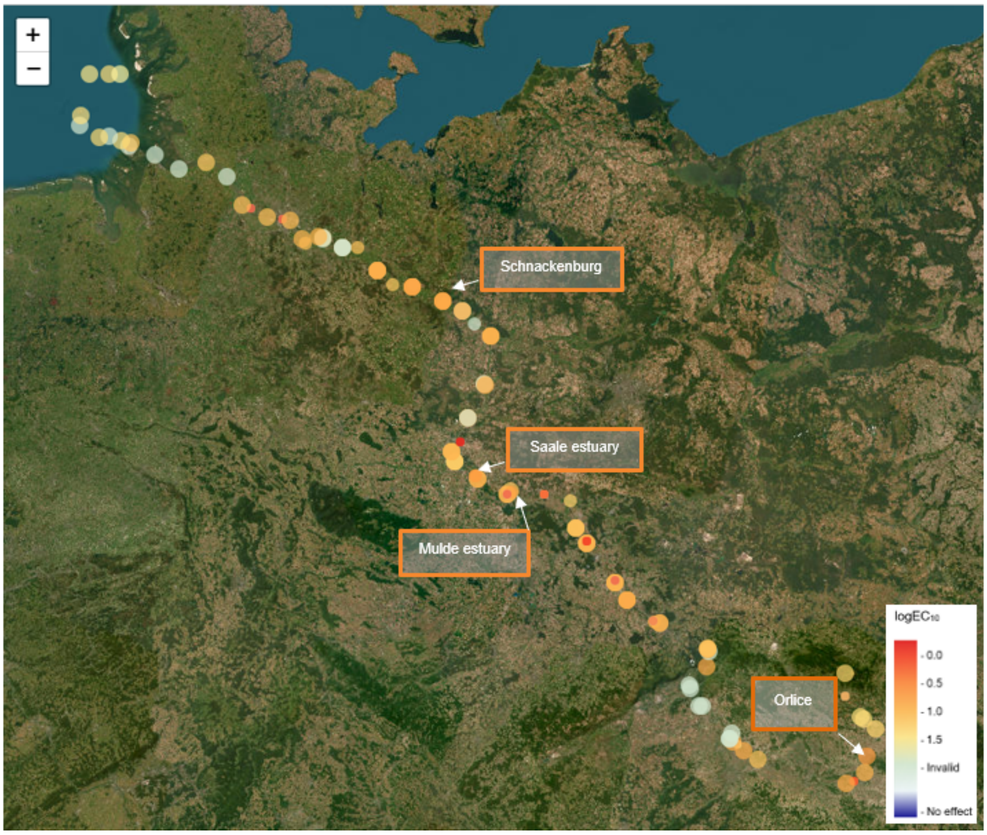

Figure S3. The logarithmic values of the EC_{10} (effect concentration 10% in Relative extraction factor) for AhR assay from Spindleruv Mlyn to the German Bight are represented graphically, with smaller circles denoting wastewater treatment plant (WWTP) influents and effluents.

An invalid (light blue) result indicates an EC_{10} exceeding the IC_{10} or no effect but cytotoxicity, while the dark blue dots signify no effect and no cytotoxicity up to a REF of 100.

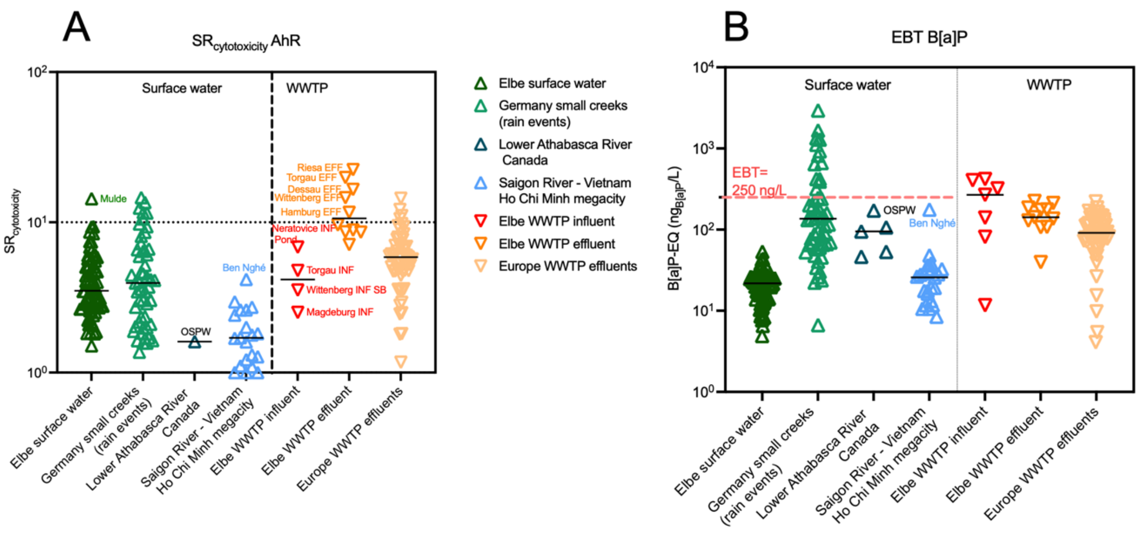

Figure S4. Comparison of effect data with literature.

A) The specificity ratio $SR_{cytotoxicity}$ of the AhR CALUX of the Elbe study compared with German small creeks Lee et al.[7], oil sands process-affected water Barrow et al.[11], impact of megacities on the Saigon river Caracciolo et al.[12] and European WWTP (waste water treatment plant) effluents Finckh et al.[13] to compare the different sampling sites. B) The B[a]P-EQ levels of the Elbe study were compared with the B[a]P-EQ of the studies described in Section A, relative to the effect-based trigger value (EBT) of B[a]P-EQ for AhR CALUX of 250 ng/L Escher et al. [14] The mean is represented by a black line on the graph.

Hotspots for the oxidative stress response mediated activity

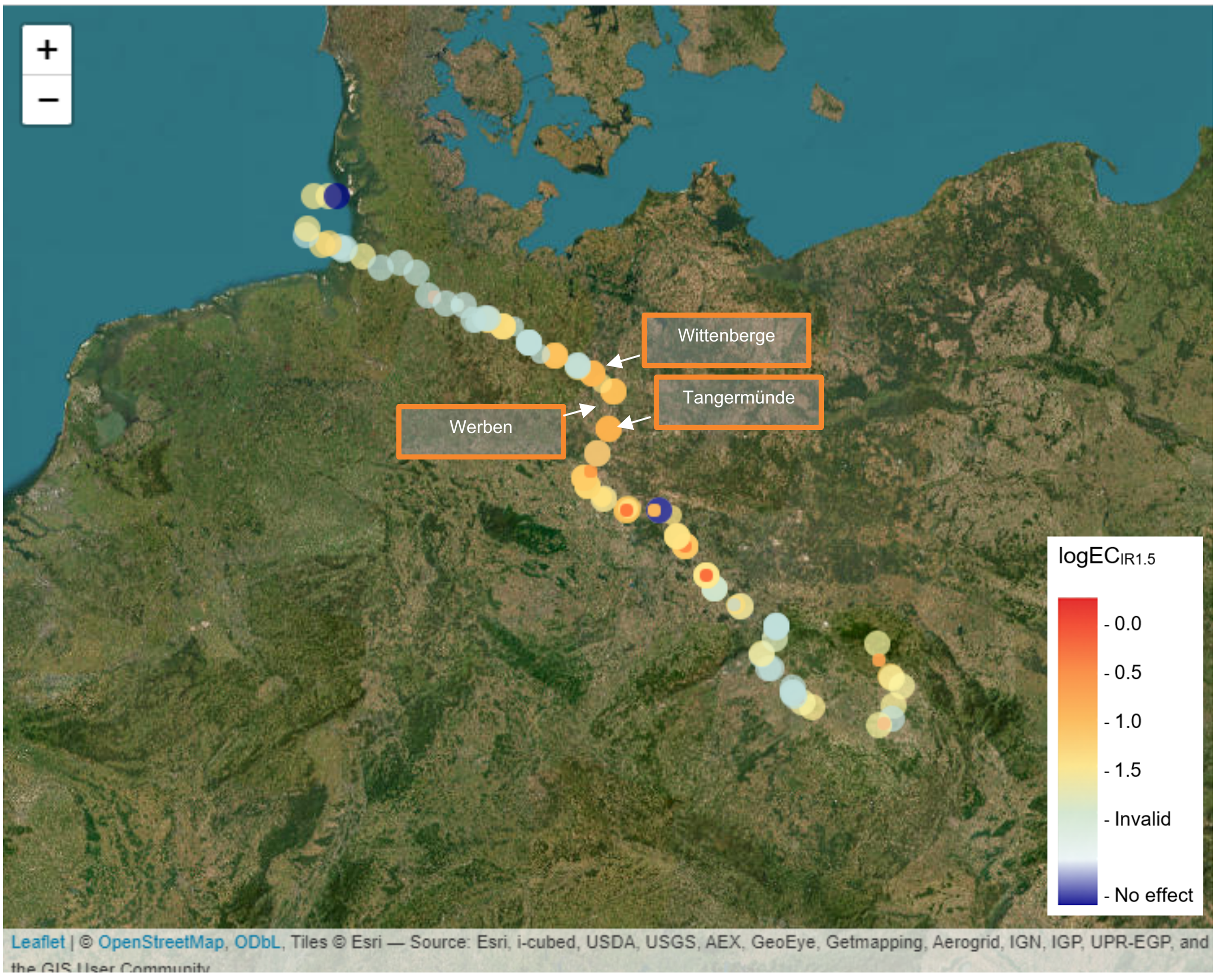

Figure S5. Oxidative stress response (AREc32) along the Elbe.

Logarithmic values of the $EC_{IR1.5}$ (effect concentration causing an induction ratio of 1.5, in REF (relative extraction factor)) for the AREc32 bioassay from Spindleruv Mlyn to the German Bight are represented graphically, with smaller circles denoting wastewater treatment plant (WWTP) influents and effluents. An invalid (light blue) result indicates an EC_{10} exceeding the IC_{10} or no effect but cytotoxicity, while the dark blue dots signify no effect and no cytotoxicity up to a REF of 100.

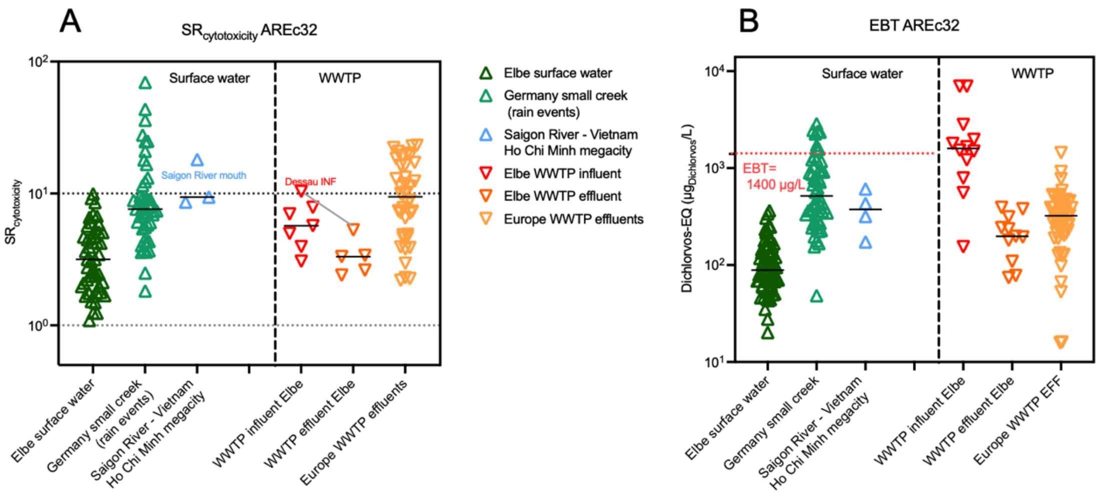

Figure S6. Comparison of oxidative stress response (AREc32) of the Elbe samples with literature.

A) The specificity ratio $SR_{cytotoxicity}$ of the AREc32assay of the Elbe study compared with German small creeks Lee et al.[7], oil sands process-affected water Barrow et al.[11], impact of megacities on the Saigon river Caracciolo et al.[12] and European WWTP (waste water treatment plant) effluents Finckh et al.[13] to compare the different sampling sites. B) The Dichlorvos-EQ (equivalence concentration) levels of the Elbe study were compared with the Dichlorvos-EQ of the studies described in Section A, relative to the effect based trigger value (EBT) of 1400 µg/L Neale et al.[14]. The mean is represented by a black line on the graph.

Hotspots for the estrogen receptor α -mediated activity

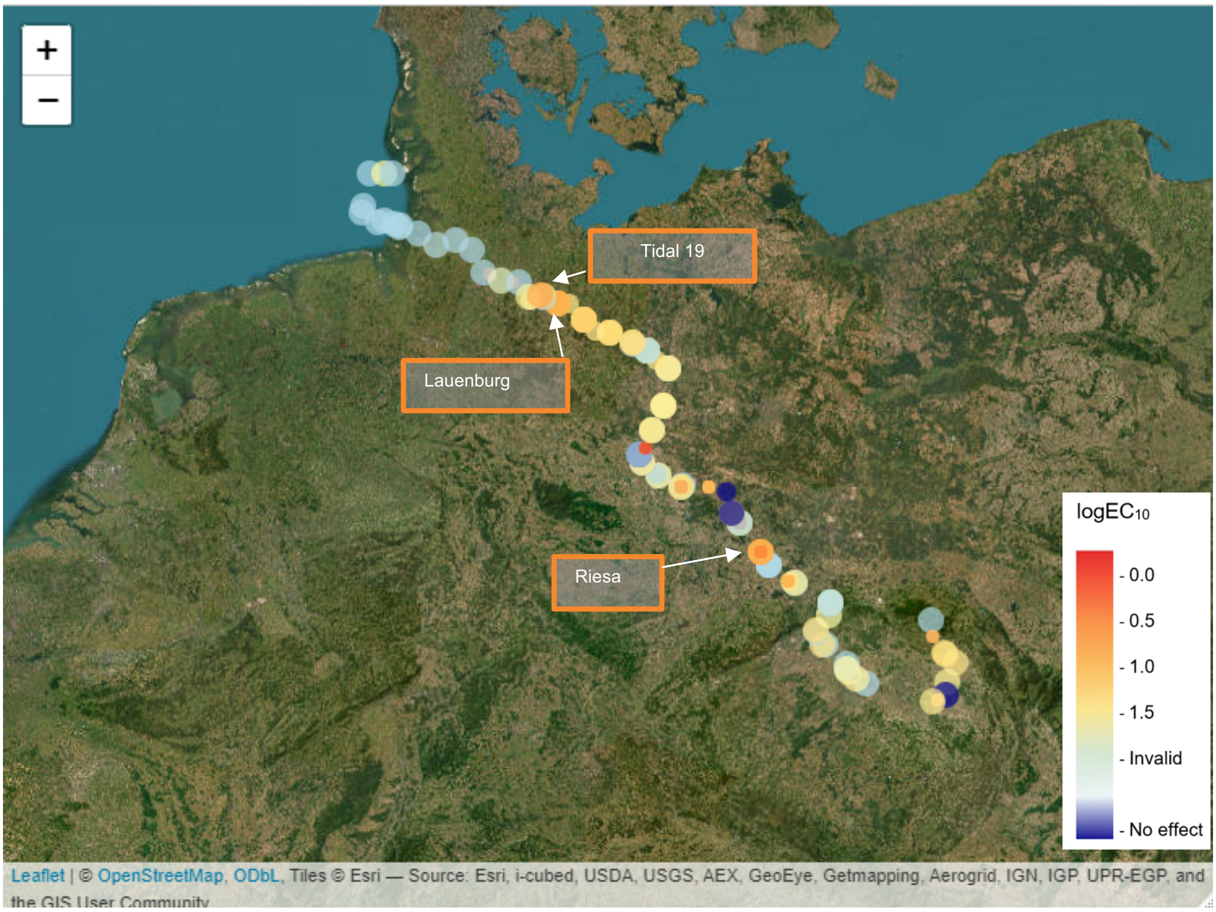

Figure S7. logarithmic values of the EC_{10} (effect concentration 10% in REF (relative extraction factor)) for the ERα assay from Spindleruv Mlyn to the German Bight are represented graphically, with smaller circles denoting wastewater treatment plant (WWTP) influents and effluents.

An invalid (light blue) result indicates an EC_{10} exceeding the IC_{10} or no effect but cytotoxicity, while the dark blue dots signify no effect and no cytotoxicity up to a REF of 100.

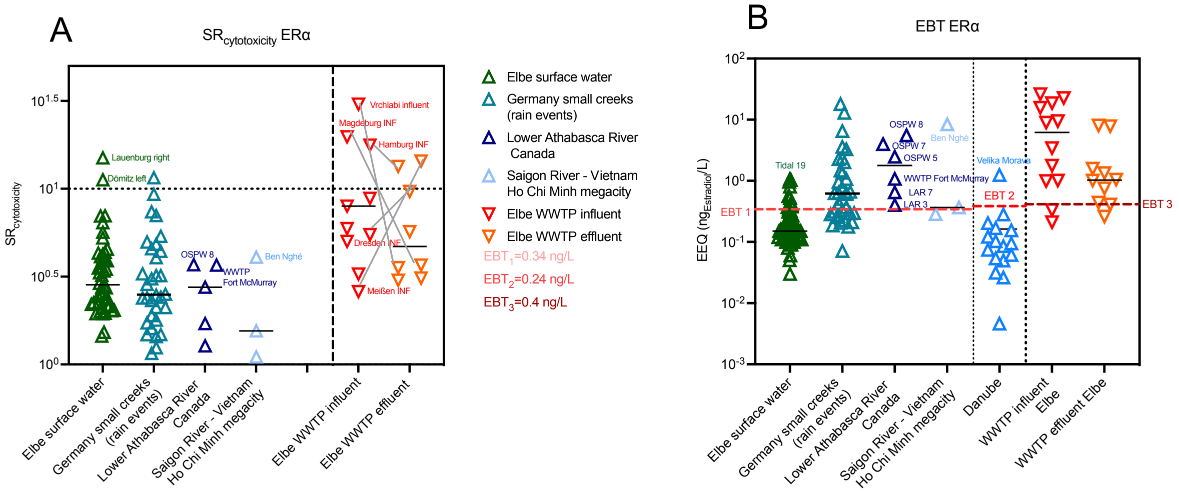

Figure S8. Comparison of EEQ of the Elbe with literature.

A) The specificity $SR_{cytotoxicity}$ of the ERα GeneBLAzer of the Elbe study compared with German small creeks Lee et al.[7], oil sands process-affected water Barrow et al.[11], impact of megacities on the Saigon river Caracciolo et al.[12] B) The EEQ (estradiol equivalent concentration) of the Elbe study were compared with the EEQ of compared with the studies described in A) and the Joint Danube Survey (cell line MELN from the study Neale et al.[15], EBT 0.37 ng/L from Escher et al.[16] to compare different sampling sites, relative to the effect based trigger value (EBT) for ERa GeneBLAzer with EBT-EEQ of 0.34 ng/L Neale et al.[14]. The mean is represented by a black line on the graph.

Hotspots for neurotoxic mediated activity in the SHSY5Y assay

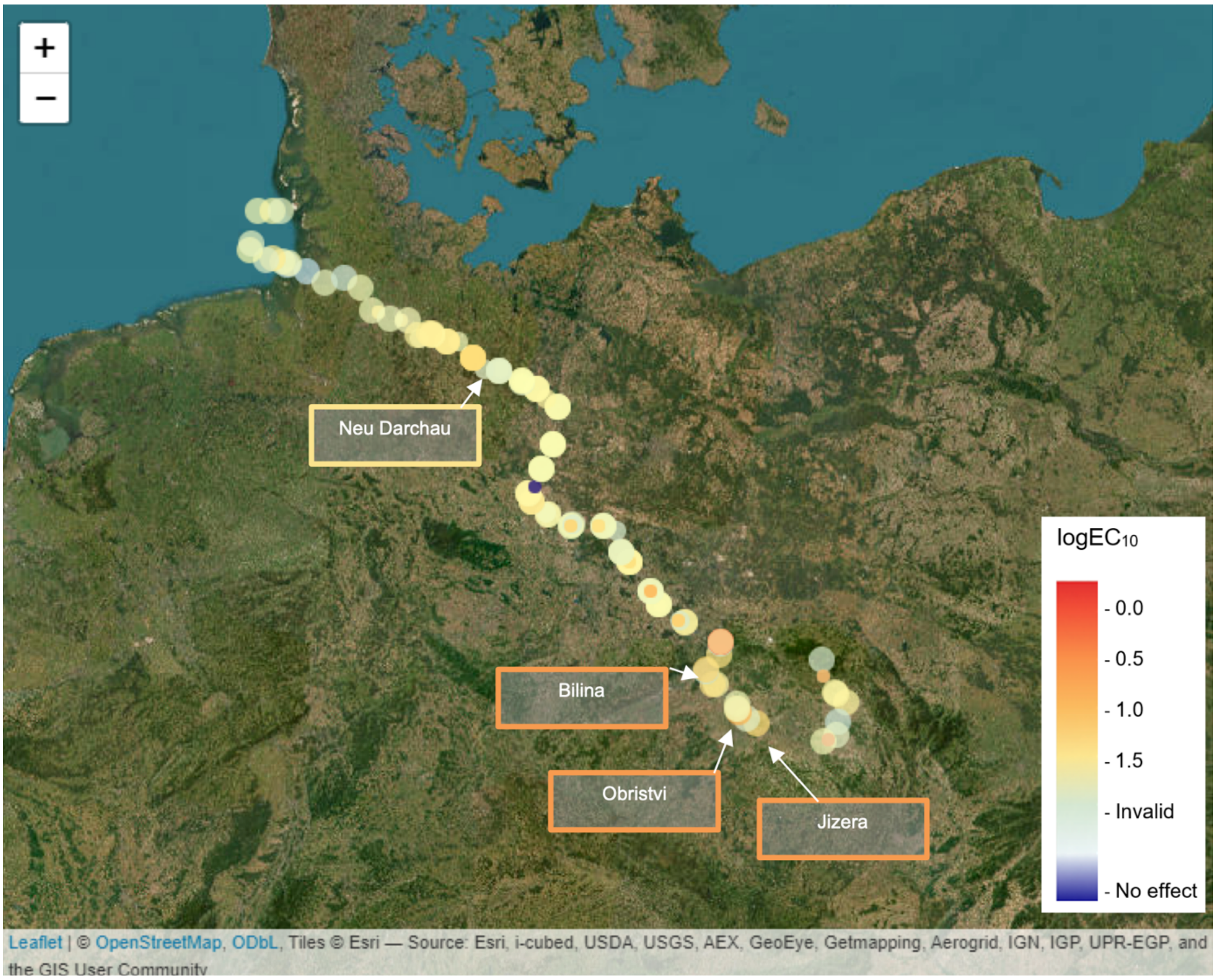

Figure S9. Neurotoxicity (SH-SY5Y) along the Elbe.

Logarithmic values of the EC_{10} (effect concentration 10% in units of REF) for the SH-SY5Y neurotoxicity assay from Spindleruv Mlyn to the German Bight are represented graphically, with smaller circles denoting wastewater treatment plant (WWTP) influents and effluents. An invalid (light blue) result indicates an EC_{10} exceeding the IC_{10} or no effect but cytotoxicity, while the dark blue dots signify no effect and no cytotoxicity up to a REF of 100.

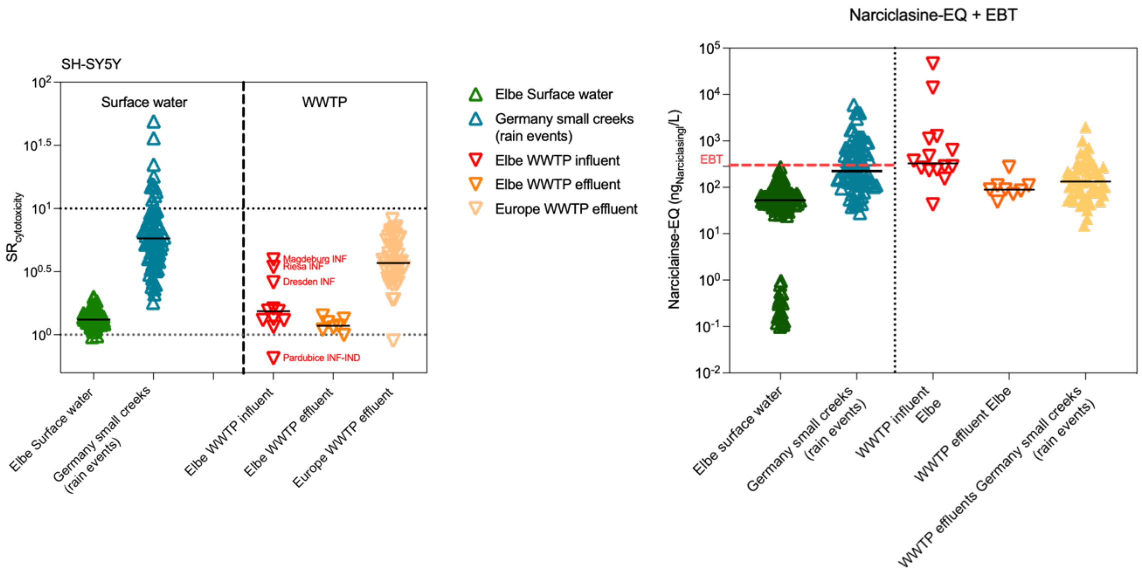

Figure S10. Comparison of neurotoxicity (SH-SY5Y) of Elbe samples with literature data.

The specificity ratio $SR_{cytotoxcitiy}$ of the neurotoxicity assay of the Elbe study compared with German small creeks Lee et al.[7], and European wastewater treatment effluents (WWTP) Finckh et al.[13] to compare the different sampling sites. The mean is represented by a black line on the graph.

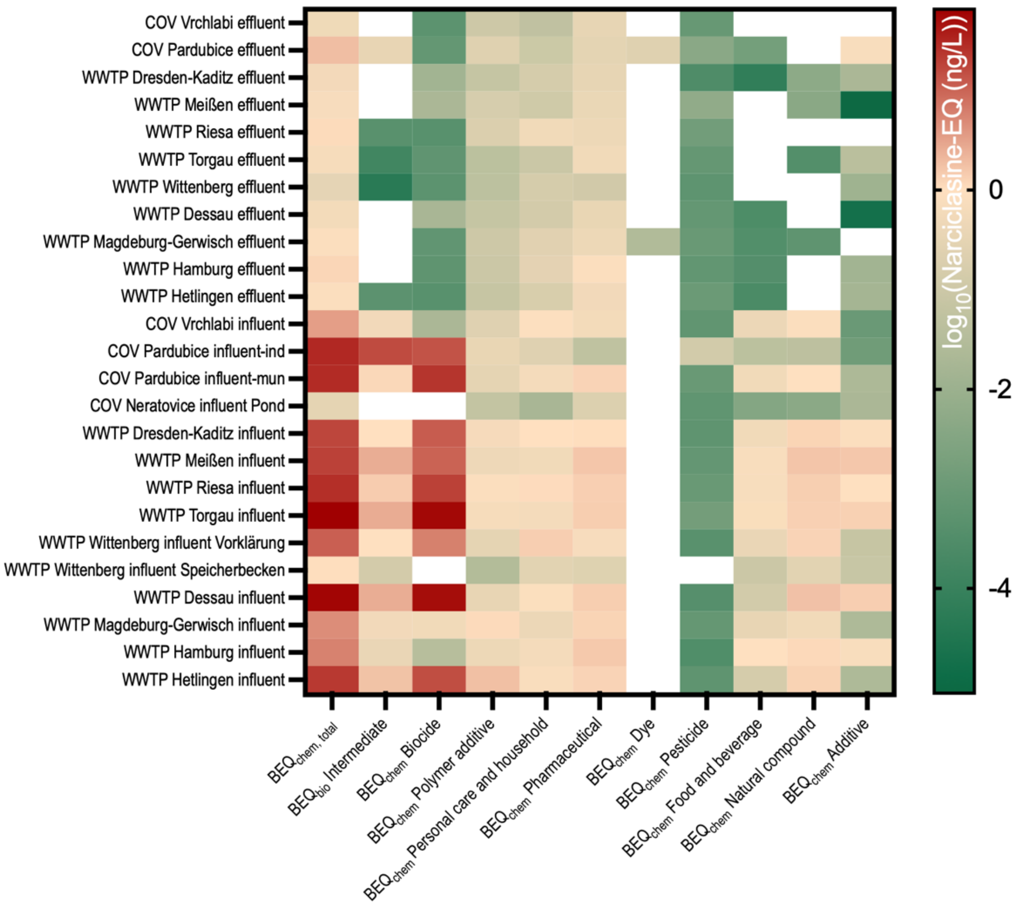

Figure S11. Categorical chemical profile BEQ_{chem} of wastewater treatment plant (WWTP) influent and effluents into the Elbe from the neurotoxicity assay. Contribution of chemical categories to the predicted mixture effect $BEQ_{chem,total}$ in Narciclasine- EQ.

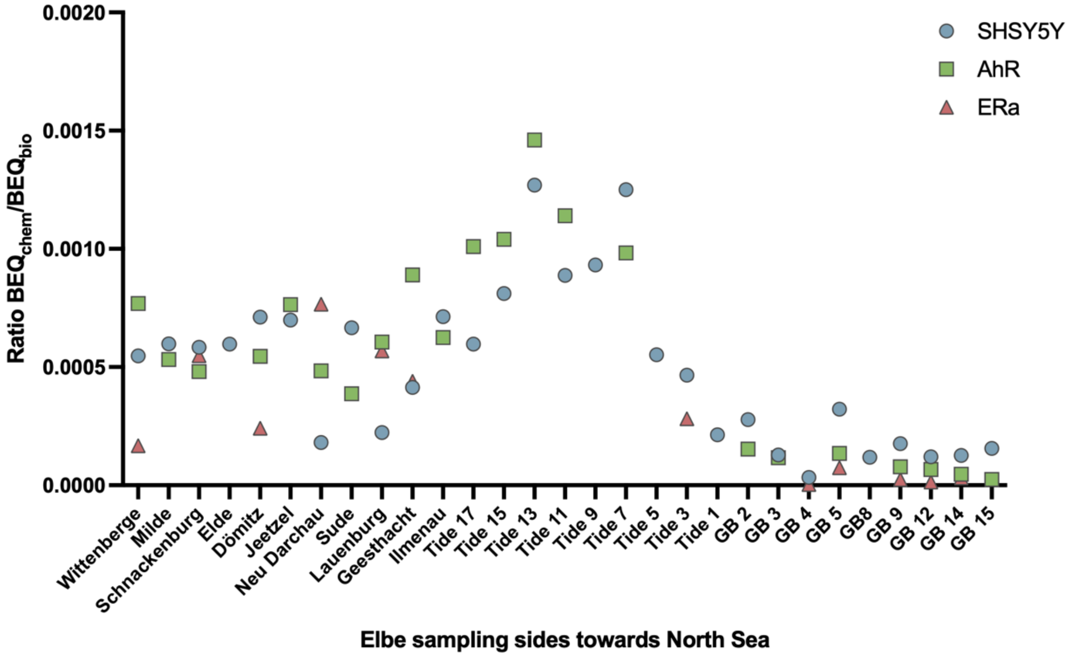

Figure 12 BEQ_{chem} to BEQ_{bio} ratio from Wittenberge to the German Bight, indicating a decrease after sampling point tidal 9.

Toxicity drivers identified in the Iceberg modeling for WWTP effluents and influents

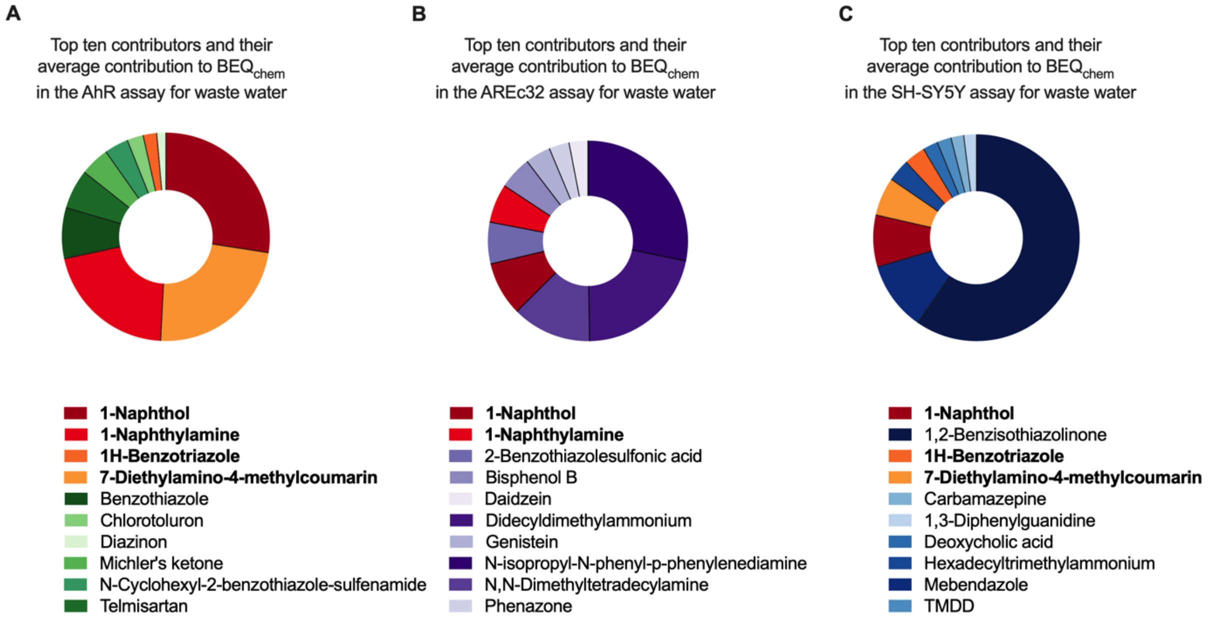

Figure S13. Toxicity driver for WWTP effluents and influents for the AhR, SHSY5Y and AREc32 assay.

References

(1) Chambers, M. C.; Maclean, B.; Burke, R.; Amodei, D.; Ruderman, D. L.; Neumann, S.; Gatto, L.; Fischer, B.; Pratt, B.; Egertson, J.; et al. A cross-platform toolkit for mass spectrometry and proteomics. *Nature Biotechnology* **2012**, *30* (10), 918-920. DOI: 10.1038/nbt.2377.
(2) Pluskal, T.; Castillo, S.; Villar-Briones, A.; Orešič, M. MZmine 2: modular framework for processing, visualizing, and analyzing mass spectrometry-based molecular profile data. *BMC bioinformatics* **2010**, *11*, 1-11. DOI: 10.1186/1471-2105-11-395.
(3) Beckers, L.-M.; Brack, W.; Dann, J. P.; Krauss, M.; Müller, E.; Schulze, T. Unraveling longitudinal pollution patterns of organic micropollutants in a river by non-target screening and cluster analysis. *Science of The Total Environment* **2020**, *727*. DOI: 10.1016/j.scitotenv.2020.138388.
(4) Schulze, T.; Mueller, E.; Krauss, M. *MZquant: Workflow for blank tagging and quantification of MZmine output files. https://git.ufz.de/wana_public/mzquant* (accessed 2024/09/24). 2021. https://git.ufz.de/wana_public/mzquant.
(5) USEPA. Definition and Procedure for the Determination of the Method Detection Limit Revision 2 EPA 821-R-16-006. https://www.epa.gov/sites/default/files/2016-12/documents/mdl-procedure_rev2_12-13-2016.pdf (accessed 2024/09/24). 2016.
(6) König, M.; Escher, B. I.; Neale, P. A.; Krauss, M.; Hilscherová, K.; Novák, J.; Teodorović, I.; Schulze, T.; Seidensticker, S.; Kamal Hashmi, M. A.; et al. Impact of untreated wastewater on a major European river evaluated with a combination of in vitro bioassays and chemical analysis. *Environmental Pollution* **2017**, *220*, 1220-1230. DOI: 10.1016/j.envpol.2016.11.011.

(7) Lee, J.; Escher, B. I.; Scholz, S.; Schlichting, R. Inhibition of neurite outgrowth and enhanced effects compared to baseline toxicity in SH-SY5Y cells. *Archives of Toxicology* **2022**, *96* (4), 1039-1053. DOI: 10.1007/s00204-022-03237-x.
(8) Brennan, J. C.; He, G.; Tsutsumi, T.; Zhao, J.; Wirth, E.; Fulton, M. H.; Denison, M. S. Development of Species-Specific Ah Receptor-Responsive Third Generation CALUX Cell Lines with Enhanced Responsiveness and Improved Detection Limits. *Environmental Science & Technology* **2015**, *49* (19), 11903-11912. DOI: 10.1021/acs.est.5b02906.
(9) Wang, X. J.; Hayes, J. D.; Wolf, C. R. Generation of a Stable Antioxidant Response Element–Driven Reporter Gene Cell Line and Its Use to Show Redox-Dependent Activation of Nrf2 by Cancer Chemotherapeutic Agents. *Cancer Research* **2006**, *66* (22), 10983-10994. DOI: 10.1158/0008-5472.Can-06-2298.
(10) Escher, B. I.; Dutt, M.; Maylin, E.; Tang, J. Y.; Toze, S.; Wolf, C. R.; Lang, M. Water quality assessment using the AREc32 reporter gene assay indicative of the oxidative stress response pathway. *Journal of Environmental Monitoring* **2012**, *14* (11), 2877-2885. DOI: 10.1039/c2em30506b.
(11) Barrow, K.; Escher, B. I.; Hicks, K. A.; König, M.; Schlichting, R.; Arlos, M. J. Water quality monitoring with in vitro bioassays to compare untreated oil sands process-affected water with unimpacted rivers. *Environmental Science: Water Research & Technology* **2023**, *9* (8), 2008-2020. DOI: 10.1039/D2EW00988A.
(12) Caracciolo, R.; Escher, B. I.; Lai, F. Y.; Nguyen, T. A.; Le, T. M. T.; Schlichting, R.; Tröger, R.; Némery, J.; Wiberg, K.; Nguyen, P. D.; et al. Impact of a megacity on the water quality of a tropical estuary assessed by a combination of chemical analysis and in-vitro bioassays. *Science of The Total Environment* **2023**, *877*. DOI: 10.1016/j.scitotenv.2023.162525.
(13) Finckh, S.; Beckers, L.-M.; Busch, W.; Carmona, E.; Dulio, V.; Kramer, L.; Krauss, M.; Posthuma, L.; Schulze, T.; Slootweg, J. A risk based assessment approach for chemical mixtures from wastewater treatment plant effluents. *Environment international* **2022**, *164*, 107234. DOI: 10.1016/j.envint.2022.107234.
(14) Escher, B. I.; Neale, P. A. Effect-Based Trigger Values for Mixtures of Chemicals in Surface Water Detected with In Vitro Bioassays. *Environ.Toxicol. Chem.* **2021**, *40* (2), 487-499. DOI: 10.1002/etc.4944.
(15) Neale, P. A.; Ait-Aissa, S.; Brack, W.; Creusot, N.; Denison, M. S.; Deutschmann, B.; Hilscherová, K.; Hollert, H.; Krauss, M.; Novak, J. Linking in vitro effects and detected organic micropollutants in surface water using mixture-toxicity modeling. *Environmental Science & Technology* **2015**, *49* (24), 14614-14624. DOI: 10.1021/acs.est.5b04083.
(16) Escher, B. I.; Aït-Aïssa, S.; Behnisch, P. A.; Brack, W.; Brion, F.; Brouwer, A.; Buchinger, S.; Crawford, S. E.; Du Pasquier, D.; Hamers, T.; et al. Effect-based trigger values for in vitro and in vivo bioassays performed on surface water extracts supporting the environmental quality standards (EQS) of the European Water Framework Directive. *Science of The Total Environment* **2018**, *628-629*, 748-765. DOI: 10.1016/j.scitotenv.2018.01.340.
